# Climate change is poised to alter mountain stream ecosystem processes via organismal phenological shifts

**DOI:** 10.1073/pnas.2310513121

**Published:** 2024-03-18

**Authors:** Kyle Leathers, David Herbst, Guillermo de Mendoza, Gabriella Doerschlag, Albert Ruhi

**Affiliations:** ^a^Department of Environmental Science, Policy, and Management, University of California, Berkeley, CA 94720; ^b^Sierra Nevada Aquatic Research Laboratory, University of California, Santa Barbara, CA 93106; ^c^Institute of Biology and Earth Sciences, Pomeranian University in Słupsk, Słupsk 76-200, Poland

**Keywords:** climate change, phenology, ecosystem processes, mountain streams, low flow

## Abstract

Climate change is affecting the phenology of organisms and ecosystem processes across a wide range of environments. However, the links between organismal and ecosystem process change in complex communities remain uncertain. In snow-dominated watersheds, snowmelt in the spring and early summer, followed by a long low-flow period, characterizes the natural flow regime of streams and rivers. Here, we examined how earlier snowmelt will alter the phenology of mountain stream organisms and ecosystem processes via an outdoor mesocosm experiment in stream channels in the Eastern Sierra Nevada, California. The low-flow treatment, simulating a 3- to 6-wk earlier return to summer baseflow conditions projected under climate change scenarios in the region, increased water temperature and reduced biofilm production to respiration ratios by 32%. Additionally, most of the invertebrate species explaining community change (56% and 67% of the benthic and emergent taxa, respectively), changed in phenology as a consequence of the low-flow treatment. Further, emergent flux pulses of the dominant insect group (Chironomidae) almost doubled in magnitude, benefitting a generalist riparian predator. Changes in both invertebrate community structure (composition) and functioning (production) were mostly fine-scale, and response diversity at the community level stabilized seasonally aggregated responses. Our study illustrates how climate change in vulnerable mountain streams at the rain-to-snow transition is poised to alter the dynamics of stream food webs via fine-scale changes in phenology—leading to novel predator–prey “matches” or “mismatches” even when community structure and ecosystem processes appear stable at the annual scale.

## Significance

In mountain regions globally, climate change is reducing snowpack, advancing snowmelt, and altering environmental regimes of rivers born in these elevations. Here, we conducted an experiment simulating end-of-century vs. current flow regimes in Sierra Nevada mountain streams to examine impending shifts in biodiversity and ecosystem processes. Early snowmelt destabilized stream epilithic biofilm metabolism and altered key ecosystem functions such as insect production and emergence, via shifts in community composition, structure, and phenology (i.e., timing of development). Notably, some processes showed sensitivity to climate change on fine timescales, with implications for predator–prey synchrony. As climate continues to change quickly in high-altitude mountain ecosystems, the resilience of stream ecosystem functions may hinge on the presence of diverse ecological communities.

Recent climate shifts in temperature and precipitation patterns have already altered the phenology of many organisms (1, 2). Climate warming has changed the timing of key life history events such as hatching, migration, mating, blooming, and death in a wide variety of plants and animals (3). These changes may benefit individual species via extended growing seasons and resource pulses; or harm them via stress, habitat contraction, and spatio-temporal mismatches between energy needs and food availability (4, 5). Mounting evidence supports that even phenological shifts of individual species can impact ecosystem processes at large scales. For example, milder winters have delayed mortality of mountain pine beetles (*Dendroctonus ponderosae*), enabling range expansion and causing widespread tree mortality that has transformed forests from being carbon sinks to sources (6). Similarly, warmer springs have advanced ephemeral plant flowering but not pollinator emergence, ultimately reducing production (7). However, many ecosystem processes (e.g., primary production, secondary production, and cross-ecosystem subsidies) often depend on many species. While it is typically assumed that phenological shifts can alter ecosystem processes, few studies have examined this question in complex, multi-trophic systems (8, 9).

Understanding the link between phenological change and ecosystem process change is particularly crucial in streams and rivers because freshwater ecosystems are highly sensitive to environmental change (10). Climate change has disproportionately eroded freshwater species populations (11), and extinction rates for freshwater organisms under future climate change are expected to be an order of magnitude higher than for marine and terrestrial counterparts (10, 12). This high vulnerability is due to the fragmented nature of freshwater habitat, the climate sensitivity of thermal and hydrologic regimes (10, 12), and the dominance of ectotherms in freshwater food webs (13). Despite the high potential for climate-driven phenological shifts in fresh waters, it is uncertain how whole communities may respond to warming—and whether phenological change may alter the ecosystem processes that these organisms control (14).

Among freshwater ecosystems, small streams in snow-dominated catchments are particularly vulnerable to climate change (15). In mountain ranges where snow is the dominant form of precipitation (e.g., in California’s Sierra Nevada), snowmelt in the late spring and early summer constitutes the majority of annual runoff and is often followed by a period of baseflow conditions in late summer and fall (16), in which streams are sustained by groundwater. Here, we use the general term low flow in place of baseflow to describe low discharge levels during the dry season (17). Climate change is predicted to reduce snowpack and advance snowmelt, which will extend summer low flow duration by up to 2 mo by the end of the century, increasing the overlap between periods of low flow and peak air temperature (18). Climate change has already altered snowmelt in mid-elevation mountain ranges globally, by decreasing snowpack and shifting the rain-to-snow transition zone (19). Some impacts of extended summer low flows on stream biodiversity and ecosystem processes, like fish population declines, often occur rapidly via physiological stress when flow drops below a threshold (20); in contrast, other responses may be cumulative [e.g., the accumulation of cyanobacteria in biofilm (21)] and may thus only be noticeable after a period of time. However, few studies have examined the immediate vs. delayed effects of low flows on stream biodiversity and ecosystem processes using frequent temporal monitoring. Such an approach is costlier than before*-*after experimental designs but may reveal the scales and mechanisms driving ecological change more precisely (20).

One key impact of earlier, extended summer low flow conditions in small streams is that low flows may accelerate climate-driven warming via reduced thermal buffering. Warming can shift community composition and structure by replacing species adapted to cold, well-oxygenated waters (cold stenotherms) with those from warmer environments (eurytherms) (22). Warming also controls key ecosystem processes and often increases ecosystem-level primary production and respiration rates (23, 24). Because water temperature controls metabolic rates of ectotherms, warming is expected to speed up aquatic insect growth rates and development, potentially advancing the timing of metamorphosis and emergence of adult, flying insects. In turn, changes in the timing and/or magnitude of emerging insects could affect foraging behavior of riparian birds, lizards, and bats, which often rely on emerging aquatic insects as a resource pulse (25). However, we note here that temperature-driven changes in secondary production are not well understood. Theory predicts that warming should not affect secondary production, given the approximately opposed effects that warming should have on community biomass (by shrinking mean body size of species) and turnover rates (by accelerating them) (26, 27). Empirical tests have provided mixed support for this expectation, owing to variation in species thermal preferences (28) and basal resources responding to warming (29). The link between warming-driven community change in a stream food web and changes in ecosystem processes has become a recent focus of research (24), and it could be greatly advanced by experiments with more complex, realistic assemblages.

Here, we sought to test how climate-induced, extended summer low flow conditions, simulating an end-of-century hydroclimate of reduced snowpack and earlier snowmelt (18) will alter the phenology of mountain stream organisms—and the ecosystem processes that these organisms control. In contrast to most research on the topic, focused on the effects of flow magnitude (30–33), here, we focused on the effects of an earlier snowmelt-driven flow recession associated with a longer summer low flow period (i.e., low-flow timing and duration) to better examine ecological impacts arising from phenological change. We broadly hypothesized that this climate change-induced flow regime change would alter the whole food web—from epilithic biofilm metabolism to stream invertebrate production and emergence, primarily through increases in water temperature (3, 4). However, in agreement with recent findings on thermal response diversity [i.e., different species respond in different directions and/or magnitudes to temperature change (27)], we also expected the community-level responses to be buffered against change, relative to population-level responses.

**Fig. 1.**
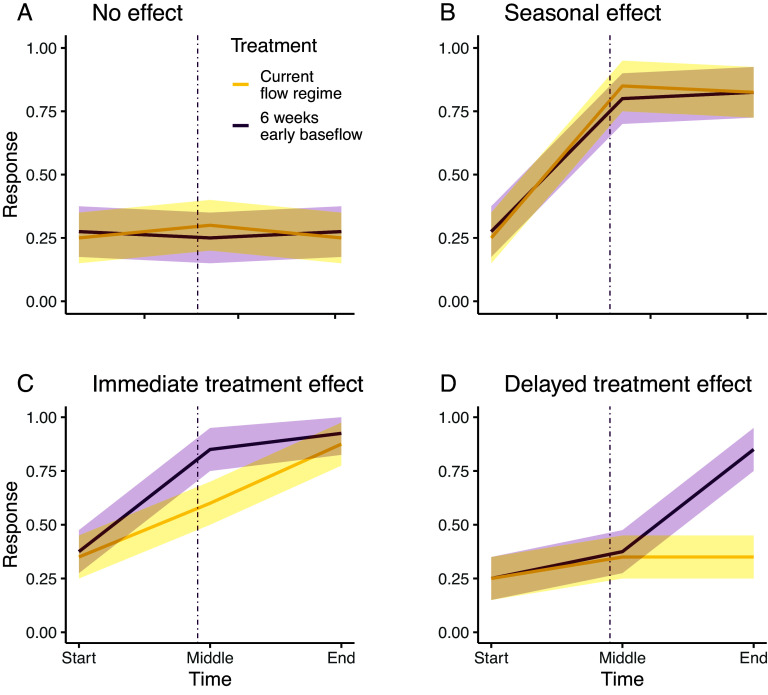
Potential ecological responses to low-flow treatment. Each pair of solid lines represents a potential ecological response to the low-flow treatment, arising from comparing treatment trajectories at the start, middle, and end of the experiment. The vertical dashed line indicates the onset of summer low-flow. (*A*) No discernible change over the study period would support the absence of seasonal or treatment effects. (*B*) Seasonal effects would be evidenced by both treatments exhibiting similar shifts over time. (*C*) An immediate treatment effect would be evidenced by treatments differing significantly at the onset of treatment differences in the *middle* period, but not at the start. This difference may be temporary (as shown here), or sustained through the end of the experiment. (*D*) A delayed treatment effect would be evidenced by no shift occurring immediately after treatment onset, but rather at the end of the experiment. In our study, we predicted that differences in the timing of the onset and duration of summer low flow would cause immediate or delayed changes in our ecological responses. Onset of summer low flow in the 6-wk treatment marks the transition between the start and middle periods, while onset of summer low flow for the Current treatment marks the transition between the middle and end periods. See *SI Appendix,* Table S1 for details on how statistical tests provide support for each of the four potential responses.

Specifically, we predicted that extended summer low flow would: 1) increase water temperature and biofilm respiration—altering the rates and balance of biofilm metabolism; 2) advance phenology and secondary production of stream invertebrates, but not change production at the seasonal scale due to stabilizing mechanisms (e.g., response diversity); and 3) advance cross-ecosystem subsidies of emergent stream invertebrates, which could be consequential if overlap shifts between peak resource availability and peak demand by riparian predators. Notably, while some of these changes may be apparent immediately, others may build over time (Fig. 1).

In order to test our predictions, we subjected nine flow-through outdoor stream mesocosms (*SI Appendix,* Fig. S1) in California’s Sierra Nevada to three flow regime treatments: a flow regime based on historic average conditions (Current treatment), a mitigated climate change scenario where streamflow returns to summer low flow levels 3 wk earlier than currently (3-wk treatment), and an unmitigated climate change scenario where summer low flow begins 6 wk earlier than currently (6-wk treatment) (18). Over the course of a season, we regularly measured epilithic biofilm production and community composition, production, and emergence of benthic and emergent stream invertebrates. We examined support for seasonal, immediate, and delayed ecological responses to the low-flow treatment (Fig. 1) by quantifying changes in magnitude and phenology for each response variable. Specifically, we combined study period (i.e., start, middle, and end) with treatment (Current, 3-wk, 6-wk), creating a variable that captures both timing and treatment effects (i.e., period–treatment; Fig. 1). This allows us to examine how low-flow treatments altered phenology. When period–treatment had an effect, we ran directed pairwise tests to identify which response type occurred (i.e., a seasonal effect, an immediate treatment effect, or a delayed treatment effect; *SI Appendix*, Table S1). In addition to testing each prediction, we ran a piecewise structural equation model to identify causal pathways connecting extended low flows to our ultimate end point in the food web: aquatic insect benthic production and emergence, a critical cross-ecosystem subsidy connecting streams to riparian ecosystems (34, 35).

## Results

### Effects of Earlier, Extended Low Flows on Abiotic Variables and Epilithic Biofilm.

The early low-flow treatment drove changes in water temperature, including a 4.6 to 7.5 °C increase in maximum water temperature with the onset of summer low flow (*F*_8,14_ = 120.3, *P* < 0.001; *SI Appendix*, Figs. S2–S4 and Table S2). We also observed a 2.6 °C increase in the diel range of water temperature in the 6-wk treatment with the onset of summer low flow, as maximum temperatures were higher and minimum temperatures were lower (*SI Appendix,* Fig. S5). Dissolved oxygen declined seasonally (*F*_8,13_ = 14.18, *P* < 0.001), but channels remained well oxygenated throughout the experiment (*SI Appendix*, Fig. S6 and Table S3).

**Fig. 2.**
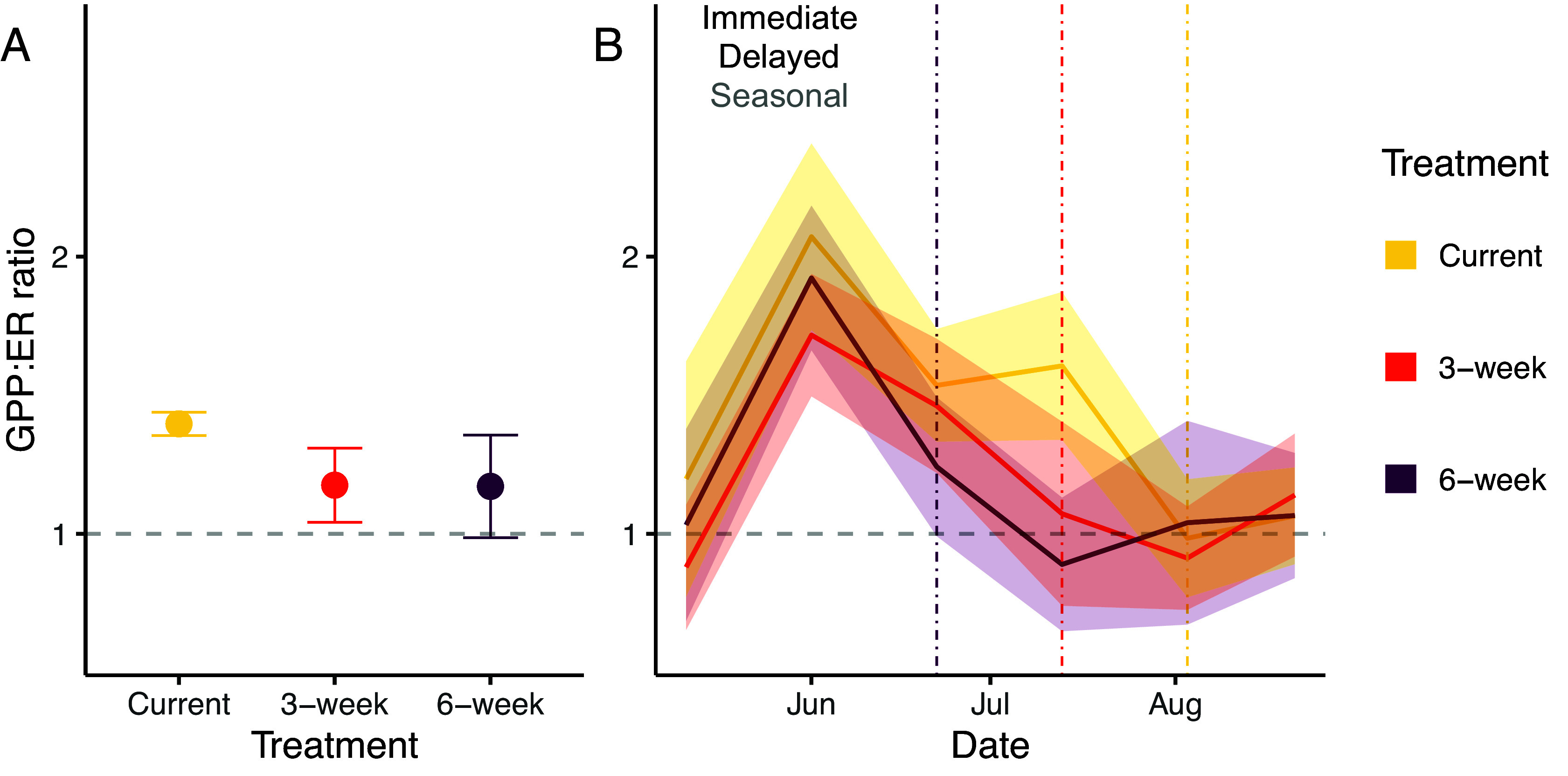
Earlier, extended low flows altered epilithic biofilm metabolism. (*A*) Mean epilithic biofilm GPP:ER ratio (±SE) for the cumulative seasonal GPP and ER. The horizontal dashed gray line represents an equilibrium between GPP and ER (*N* = 9). (*B*) Mean GPP:ER ratio (±SE) at each sampling date. The 6-wk treatment GPP:ER in the middle of the experiment was significantly lower than that of the Current treatment (*t*_45_ = −3.22, *P* = 0.011). The Current treatment was the only treatment in which ratios significantly declined between the middle and end periods, constituting a delayed effect (*t*_45_ = 3.43, *P* = 0.011). Vertical dashed lines represent the onset of summer low flow, colored by treatment. The three potential response types (immediate treatment effect, delayed treatment effect, seasonal effect) are listed, and are colored black when supported (see conceptual framework in Fig. 1, and *SI Appendix,* Table S1 for how statistical tests connect with each response type). Breakpoints in the time series plot denote each sampling event.

Low-flow timing affected the estimated production and respiration rates of epilithic biofilm—the base of production of our stream food web, which lacked macrophytes or plankton. Cumulative seasonal epilithic biofilm gross production to respiration ratios (GPP:ER) did not differ significantly by treatment, but there was an immediate decline in GPP:ER ratios with low-flow treatment (Fig. 2). The GPP:ER ratio responded to period–treatment (*F*_8,41_ = 3.307, *P* = 0.005) and was 32.2% lower for the 6-wk treatment in the middle of the experiment compared to the Current treatment, partially supporting our prediction (*SI Appendix,* Table S4). ER increased immediately for the 6-wk treatment in the middle period (*F*_8,41_ = 3.707, *P* = 0.002), showing 77.4% higher ER levels than the Current treatment (*SI Appendix*, Fig. S7 and Table S5). GPP also responded to treatment over time (*F*_8,41_ = 2.3, *P* = 0.039), but there was no support for any of the potential response types. Overall, the low-flow treatment shifted the phenology of epilithic biofilm metabolism as expected, tipping the balance between production (GPP) and respiration (ER) toward the latter.

### Effects of Earlier, Extended Low Flows on Invertebrate Communities.

**Fig. 3.**
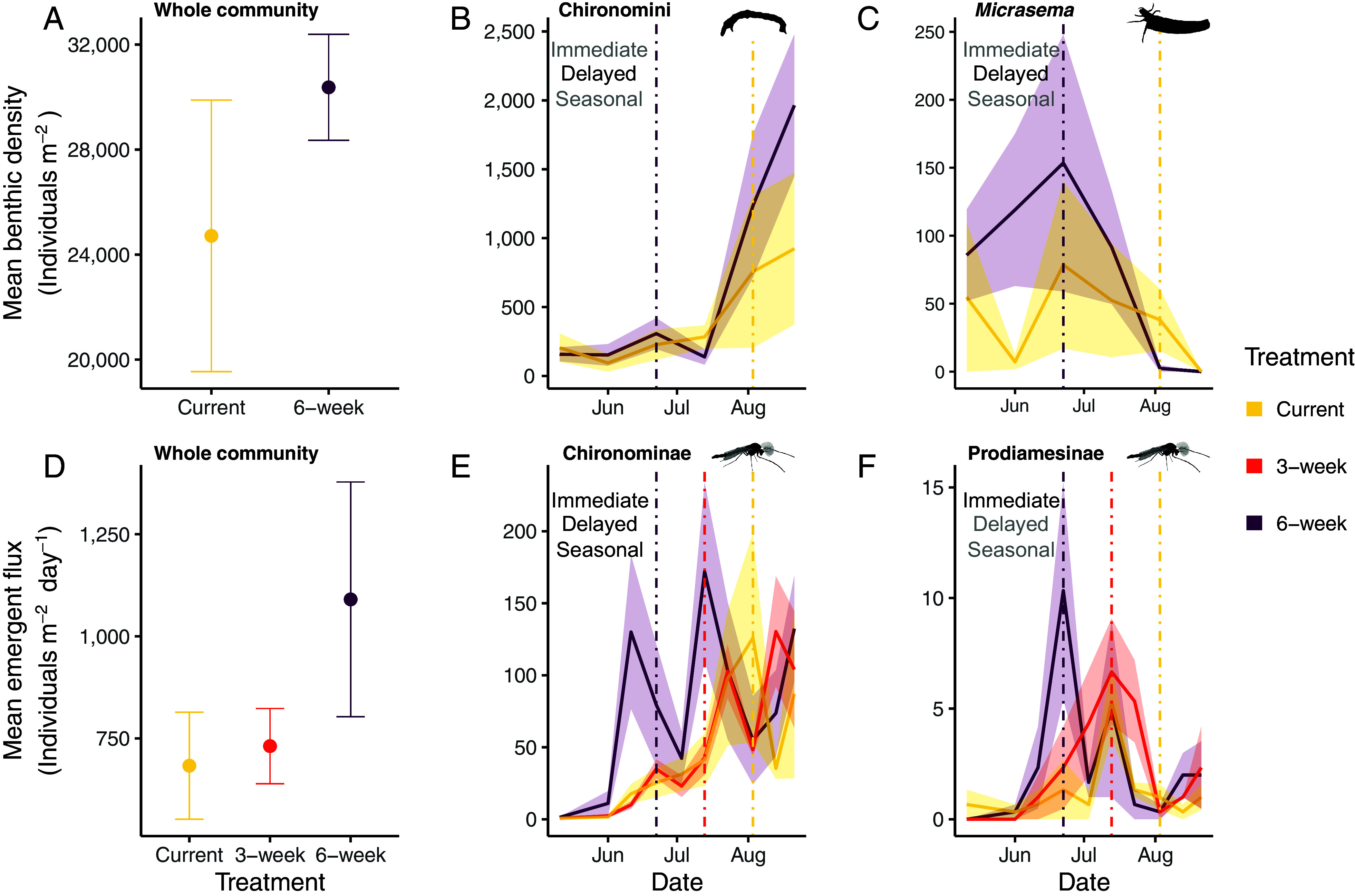
Earlier, extended low flows did not alter aggregate seasonal density or emergence, but phenological shifts occurred in several taxa. In each panel, the mean density/flux of stream invertebrates (±SE; shaded area) is represented across treatments or time. In all shown taxa, density or flux values were significantly explained by period–treatment (*P* < 0.05). The three potential response types (immediate treatment effect, delayed treatment effect, and seasonal effect) are listed, and colored black when supported (see conceptual framework in Fig. 1, and *SI Appendix,* Table S1 for how statistical tests connect with each response type). Vertical dashed lines represent the onset of summer low flow, colored by treatment. (*A*) We did not observe any significant community-level response when looking at seasonally aggregated data (*N* = 6), but (*B* and *C*) Chironomini (*F*_5,26_ = 5.267, *P* = 0.002) and *Micrasema* (*F*_5,26_ = 7.017, *P* < 0.001) had delayed increases and declines in abundance, respectively, under the low-flow treatment (*N* = 36). (*D*) We also did not observe any significant emergent community-level response when looking at seasonally aggregated data (*N* = 9), but (*E* and *F*) Chironominae (*t*_81_ = 2.97, *P* = 0.008) and Prodiamesinae (*t*_81_ = 4.89, *P* = 0.027) experienced an immediate increase in emergent flux in the middle period (*N* = 90). Chironominae in the 3-wk treatment exhibited a delayed increase (*t*_81_ = −2.40, *P* = 0.0299). Breakpoints in the time series plots denote each sampling event. Panels (*A*) and (*D*) display average values across channels under each treatment, after aggregating all samples from the experiment within each channel.

The benthic stream invertebrate community exhibited fine-scale responses to low-flow timing. Cumulative (seasonally aggregated) benthic stream invertebrate abundance did not differ by treatment (Fig. 3*A*). However, the 6-wk treatment had a delayed effect on the community due to several taxa responding to summer low flow, either by increasing or by decreasing in abundance (pseudo-*F*_5,30_ = 2.571, *P* < 0.001; Fig. 3 *B* and *C* and *SI Appendix*, Fig. S8 and Tables S6–S10). Among the taxa that significantly explained community dissimilarity, 38% of them increased and 25% decreased in abundance under low-flow treatment. Taxa with the greatest responses included Chironomini (*F*_5,26_ = 5.267, *P* = 0.002; Fig. 3*B*), *Hydroptila* (*F*_5,26_ = 15.77, *P* < 0.001), and *Micrasema* (*F*_5,26_ = 7.017, *P* < 0.001). Notably, Chironomini abundance for the 6-wk treatment at the end of the experiment was 173% higher than that in the middle of the experiment. Highly resolved taxonomy for a subset of Chironomini and Pseudochironomini support that abundance increases were driven by *Apedilum*, *Polypedilum aviceps*, and *Pseudochironomus–*taxa that tolerate warm conditions. Chironomini and *Micrasema* also experienced magnitude responses in abundance, increasing and decreasing respectively under the low-flow treatment. The subset of flow-sensitive taxa caused a delayed response at the community level (*SI Appendix,* Table S11), leading to a novel assemblage at the *end* of the season. However, we note here that the abundance of scrapers (i.e., biofilm-grazing invertebrates) did not respond to low-flow treatment (F_5,28_ = 1.512, *P* > 0.05).

Emergent stream invertebrates responded to the low-flow treatments, but, in contrast to the benthic community, they did so immediately and exhibited strong phenological change (pseudo-*F*_8,81_ = 5.728, *P* < 0.001; *SI Appendix,* Fig. S8). Among the taxa that significantly explained emergent community dissimilarity, 67% of them increased in abundance under low-flow treatment (*SI Appendix,* Table S12). Chironominae was important again in driving the shift, with its abundance increasing immediately by 147% in the middle period (*F*_8,77_ = 3.79, *P* < 0.001; Fig. 3*E*; *SI Appendix,* Table S13). Post hoc analysis supported an immediate shift in emergent community composition, based on the 6-wk community being different from the Current community (*SI Appendix,* Table S14). This composition shift reflected a phenological shift, as the 6-wk community in the middle period anticipated the assemblage at the end of the experiment in the other treatments.

**Fig. 4.**
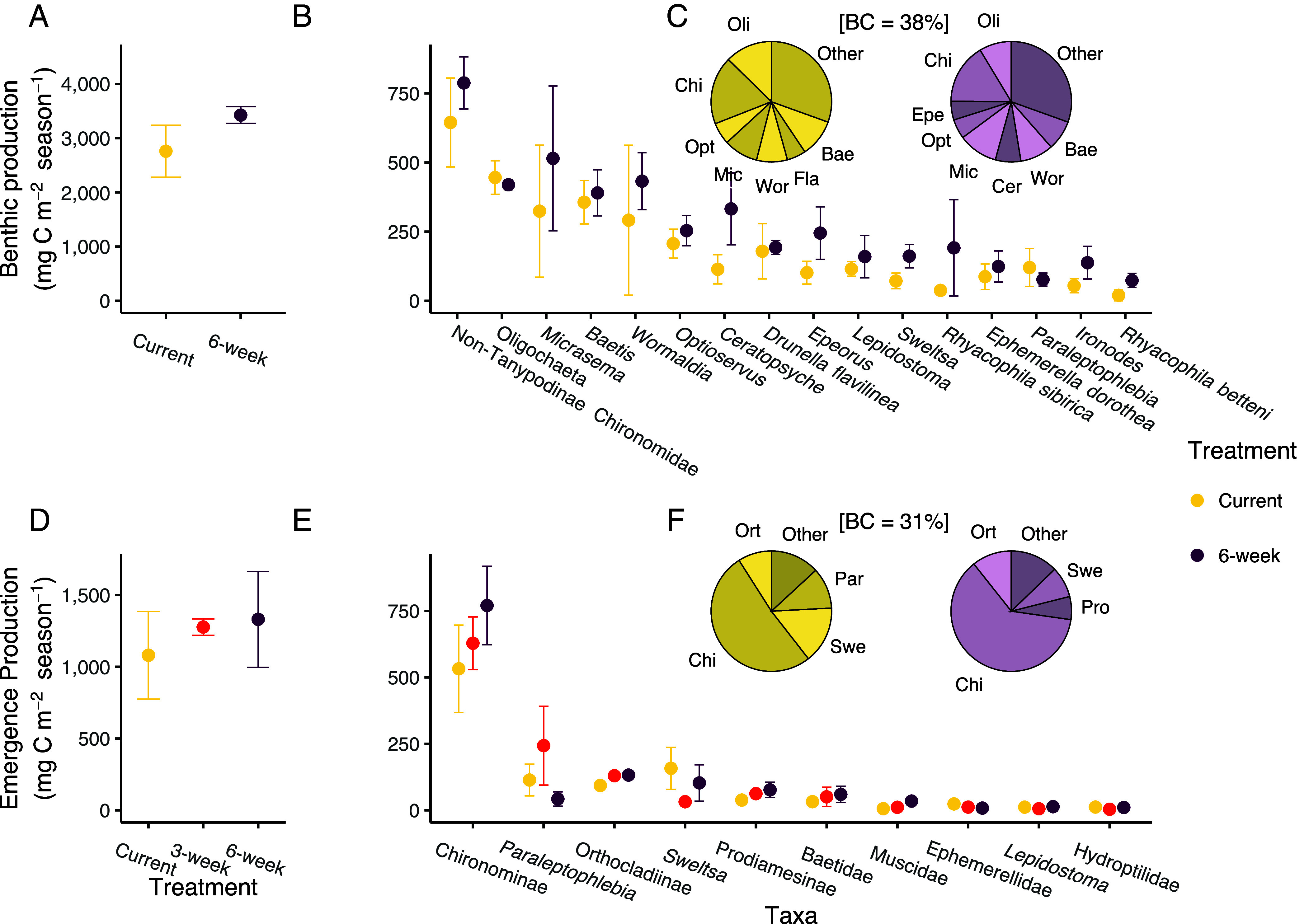
Seasonal secondary production did not differ across treatments. Mean cumulative secondary production over the study period (±SE) for: (*A*) the entire benthic community (*N* = 6); (*B*) the 16 most productive benthic taxa (*N* = 96); (*C*) the relative proportion of benthic production for taxa with more than 5% of the total community production; (*D*) the entire emergent community (*N* = 9); (*E*) the 10 most productive emergent taxa (*N* = 90); and (*F*) the relative proportion of emergent production for taxa with more than 5% of the total community production. Orthocladiinae emergence production was significantly explained by treatment (*F*_2,4_ = 12.17, *P* = 0.020). (*C* and *F*) Taxon names are abbreviated to the first three letters, and pie plots are separated by treatment for the Current and 6-wk treatments. Average Bray–Curtis dissimilarity between treatments is listed in brackets between the pie plots.

In turn, low-flow treatment did not significantly alter cumulative invertebrate secondary production (i.e., production integrated across the experiment) for either the benthic or the emergent portion of the community (Fig. 4 *A–D*). However, we did observe a wide diversity of responses across taxa, both in how their secondary production responded to low-flow treatment and in their contribution to community-wide secondary production (Fig. 4 *C* and *F*). We tested whether the lack of seasonal aggregate response in our community could be due to response diversity. We found that response diversity to change in discharge, measured as response dissimilarity, was high in our community compared to published benchmarks for response-diverse communities (36), with a median value of 2.5 (*SI Appendix,* Fig. S9). Overall, these results partially support our prediction that extended low flow conditions will shift stream invertebrate phenology. However, changes in both structure (composition) and functioning (production) were mostly fine-scale, and seasonally aggregated responses were stabilized by high community response diversity.

### Causal Pathways Connecting Low Flows to Cross-Ecosystem Resource Pulses.

**Fig. 5.**
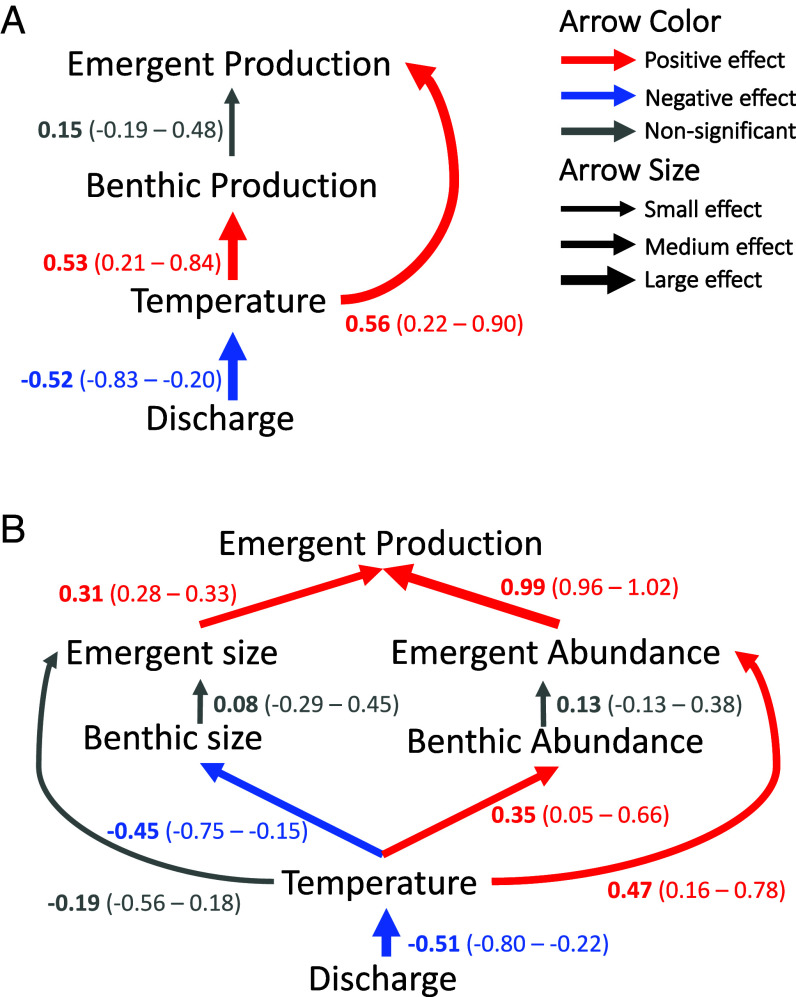
Low-flow-induced warming increased Chironomidae production and emergence via increased abundance of individuals, despite reductions in body size. (*A*) Piecewise structural equation model of the relationship between discharge and emergent Chironomidae production with temperature and benthic production linking them. Discharge has a negative relationship with water temperature (low-flow induced warming). Water temperature has positive relationships with both benthic and emergent production. (*B*) Piecewise structural equation model of the relationship between discharge and emergent Chironomidae production with mechanistic drivers linking them. Discharge has a negative effect on water temperature. Water temperature increases both benthic and emergent Chironomidae abundance but has a negative effect on benthic Chironomidae size. Emergent Chironomidae size and abundance both have positive effects on emergent Chironomidae production. Comparison of models (*A*) and (*B*) suggests that low-flow induced warming increases the emergent flux of Chironomidae midges (model *A*); this increase is realized via an increase in numerical abundance that overcompensates for their smaller body sizes, both in the benthic and emergent stages (model *B*). Both models are supported for inference based on the Fisher’s *C* statistic (Model *A*: *C*_4_ = 1.108, *P* > 0.05; Model *B*: *C*_24_ = 30.296, *P* > 0.05). Standardized estimates and associated 95% CIs are shown next to linking arrows.

Using a set of structural equation models (SEM), we examined the mechanisms connecting environmental drivers to the secondary production and subsequent emergence of the dominant aquatic insect group, Chironomidae midges (Fig. 5). This group accounted for 70% of the emergent production and 93% of the emergent abundance, thus controlling both in-stream processes and cross-ecosystem (i.e., stream-to-land) subsidies. Despite the apparent stability of Chironomidae production at the seasonal scale (see previous section), low-flow driven warming drove subseasonal variation in Chironomidae benthic and emergent production (Fig. 5*A*). This influence was realized via dual, opposing effects of temperature on Chironomidae abundance and body size (Fig. 5*B*). Specifically, warming decreased mean Chironomidae body size, but also increased their numerical abundance, with the positive effect on abundance outweighing the negative effect on body size by a factor of three (Fig. 5*B*).

**Fig. 6.**
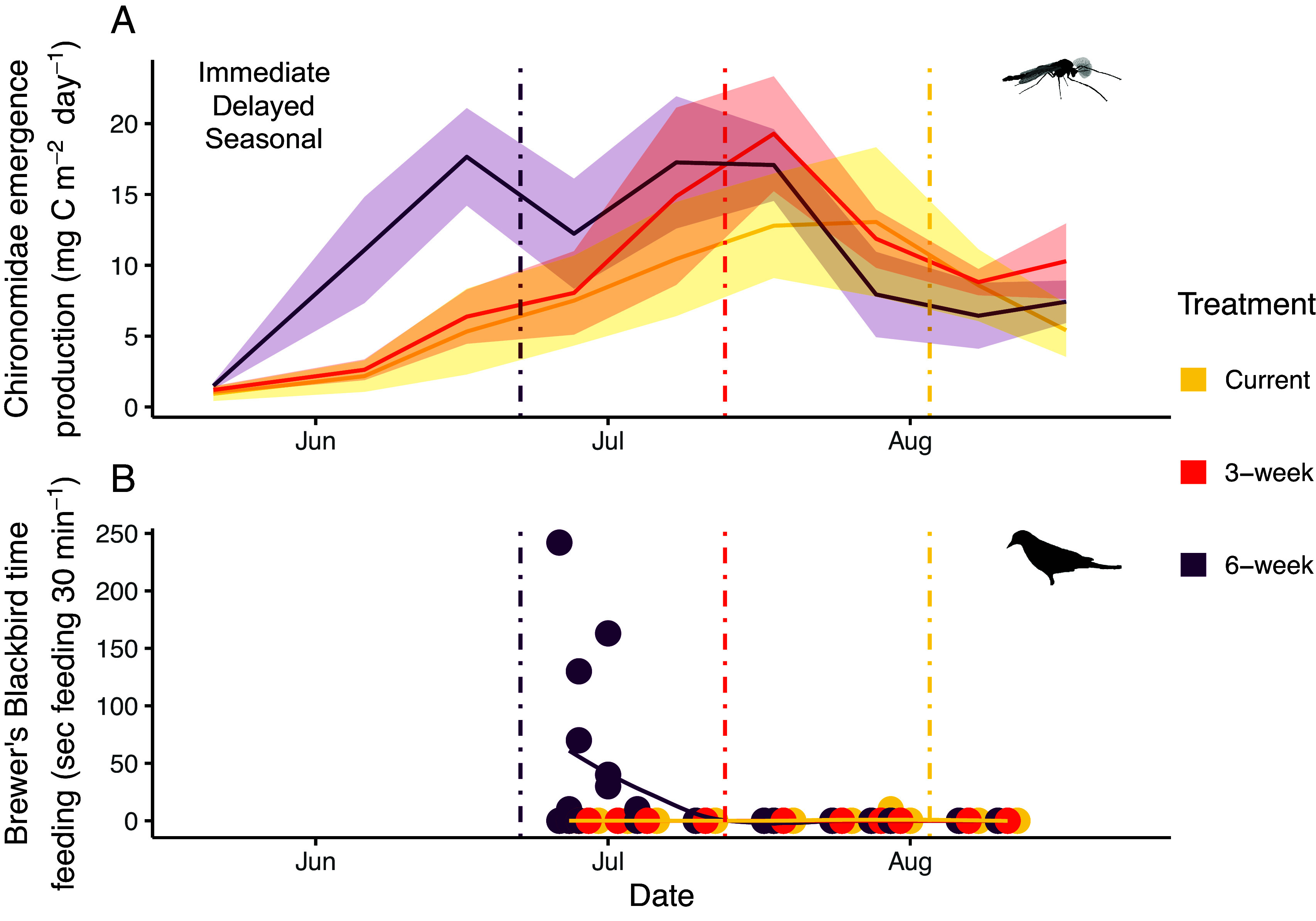
Earlier, extended low flows increased cross-ecosystem pulses. (*A*) Mean daily Chironomidae emergent production (± SE) over time (*N* = 81). The three potential response types (immediate treatment effect, delayed treatment effect, seasonal effect) are listed, and colored in black as they were all supported (see conceptual framework in Fig. 1, and *SI Appendix,* Table S1 for how statistical tests connect with each response type). Chironomidae production in the 6-wk treatment was immediately higher than in the other treatments in the middle period (*t*_72_ = 3.56, *P* = 0.004 vs. Current; *t*_72_ = 2.36, *P* = 0.039 vs. 3-wk) although all treatments showed a seasonal increase. Chironomidae production in the 6-wk treatment also showed a delayed decline in production from the middle to end periods (*t*_72_ = 2.92, *P* = 0.014). Vertical dashed lines represent the onset of summer low flow, colored by treatment. (*B*) Time in seconds that Brewer’s Blackbirds were observed in the artificial channels, over 30-min periods (*N* = 117). Brewer’s Blackbirds were present significantly more in the 6-wk treatment compared to the Current (*P* = 0.018) and 3-wk treatments (*P* = 0.01). The solid lines are smoothed conditional means using LOESS (locally estimated scatterplot smoothing) for each treatment, to assist in visualizing temporal trends.

Last, we recorded riparian bird feeding behavior to assess how low-flow treatment may have altered the behavior of a generalist predator we noticed visiting and nesting in the area, the Brewer’s Blackbirds (*Euphagus cyanocephalus*), once the experiment was underway (Fig. 6). Brewer’s Blackbirds fed on benthic macroinvertebrates in the 6-wk channels when discharge dropped to summer low-flow conditions. We began to record their behavior afterward to account for possible effects on the macroinvertebrate community. We found that emergent Chironomidae production increased in the middle period during Brewer’s Blackbirds nesting (*F*_8,68_ = 5.663, *P* < 0.001; Fig. 6). The time that Brewer’s Blackbirds spent feeding on benthic stream invertebrates in each channel was also associated with treatment (*x^2^*_2_ = 13.836, *P* = 0.001): They spent the most time in channels undergoing the 6-wk treatment (6-wk vs. Current: *P* = 0.018; 6-wk vs. 3-wk: *P* = 0.01). Brewer’s Blackbirds departed from the study site upon fledging, resulting in few observations after early July. Brewer’s Blackbirds did not have a measurable influence on the benthic or emergent macroinvertebrate community, measured either as abundance, species richness, or composition (*SI Appendix,* Table S15). Overall, these results support our prediction that our low-flow treatment will alter the phenology of aquatic-terrestrial subsidies—with changes that can be influential even if they take place at short timescales.

## Discussion

Numerous studies suggest that climate-driven phenological shifts will alter ecosystem processes (8, 9). However, few studies to date have empirically examined this link in complex, multi-trophic communities (8, 9). Here, we used an outdoor, artificial stream system in California’s Sierra Nevada to simulate future hydroclimatic conditions in mountain streams. We measured how extended summer low flows are likely to affect organism phenology, ecosystem processes, and the link between the two. We found that earlier, extended low-flow conditions will likely 1) raise water temperature, increase epilithic biofilm respiration (ER), and consequently tip the balance between epilithic biofilm production and respiration (GPP:ER); 2) advance phenology of the stream invertebrate community, even if compensatory mechanisms buffer change in production at longer, seasonal scales; and 3) alter cross-ecosystem resource fluxes by advancing emergence of key insect groups (such as Chironomidae) and by creating novel feeding opportunities for generalist riparian predators. Our findings add mechanism to the link between climate-driven phenological shifts and ecosystem process shifts (8, 24). Further, we advance the notion that ecological processes that appear insensitive to climate change at long scales can respond at finer scales—with far-reaching implications for food-web matches and mismatches (2, 37).

Our study highlights that different species responding in diverse ways to climate change stressors can stabilize community-level properties at seasonal scales. This property, often referred to as response diversity, played out at two different levels: at the population level (i.e., diverse demographic changes in size and phenology) and at the community level (i.e., dissimilar responses in species abundance across species). Notably, we found response diversity values in our complex community to be greater than those in communities previously used to illustrate a “high” response diversity level (36). Our results are consistent with observations from long-term field studies showing that even if animals have generally advanced their phenology 2.9 d per decade, substantial variation among taxa may buffer aggregate community shifts (1). While small ectotherms are generally more responsive to warming, limits to phenologic plasticity exist (1). For example, nonlinear responses to climate change can occur as a result of crossing physiological limits (e.g., critical thermal maxima), along with the local abiotic and biotic contexts interacting with each other (38). In a mesocosm study in southern England, increased temperatures altered community composition and resulting decomposition rates differently depending on the time of year (39). Additionally, temporary ecosystem process shifts due to changing phenology can cancel each other out over seasonal or yearly timescales, like we observed in the ecosystem processes we studied (e.g., secondary production). This characteristic pattern of “stability despite change” may be akin to that of “climatic debt” in climate velocity research, where a lack of community composition response can hide impending biodiversity collapse (40).

Our study suggests that streamflow, via its effects on temperature, may be the mechanism whereby climate change in mountain streams is most likely to affect organism phenology and ecosystem processes (41). Many of the low-flow effects we observed resulted from water temperature rising during low flow conditions. The GPP:ER decline we found with increasing water temperature was similar to that reported from a warming mesocosm experiment in the United Kingdom (42). In both cases, reduced GPP:ER was likely due to respiration increasing at a faster rate than GPP, based on their respective activation energies. However, low flows and high water temperatures may increase biofilm production and reduce water quality if the stream is not nutrient limited, as in our oligotrophic system (43, 44). Our methods captured biofilm production and respiration, which is only a portion of ecosystem metabolism (45). Because the light–dark bottle method excludes hyporheic metabolism and respiration from invertebrate heterotrophs, we cannot use estimates from the light–dark bottle to scale up to whole stream ecosystem metabolism. These exclusions likely underestimated ecosystem-level ER, thus preventing us from upscaling biofilm GPP:ER ratios to ecosystem-level GPP:ER ratios. Additionally, both low flows and increased water temperature have also been shown to favor small, flow-sensitive taxa like Chironomidae, which can alter overall community composition, in agreement with our results (15). We did not, however, notice a general decline in sensitive Ephemeroptera, Plecoptera, and Trichoptera taxa due to early low flows, as observed elsewhere (46). This discrepancy is likely explained by many of these taxa emerging in spring and early summer in high mountain streams. The lack of a cumulative secondary production response we observed differs from the conventional belief that low flows reduce production (20), as is often the case if flow intermittency occurs (31). However, some studies have reported that low flows may not impair, or may even slightly increase, stream invertebrate secondary production, given overcompensating increases in Chironomidae production (47). Likewise, we found that extended summer low flow is poised to increase Chironomidae production by increasing their abundance more than reducing their body size—showing that changes in ecosystem processes driven by organisms (and their phenology) may depend on a fragile balance of life-history mechanisms.

Given the dynamic nature of most responses observed, our study illustrates the need to record time-varying rather than “time-averaged” ecosystem responses to climate change. In our case, tracking responses over time allowed us to parse out immediate shifts (e.g., water temperature, biofilm production, emergence, and riparian predator feeding) from delayed or time-lagged shifts (e.g., benthic stream invertebrate community composition). These changes may connect different trophic levels—leading to novel, climate-driven “matches” or “mismatches” between resource availability and demand. While climate change leading to predator–prey mismatches is unsurprising (48), we found a novel match between peak Chironomidae emergence and Brewer’s Blackbirds nesting. Understanding when and where these new food-web links could replace current connections is important for conservation (49). However, novel matches can also be ecologically harmful. For example, advanced hatching in the moth *Agriopis aurantiaria* is increasingly coinciding with sub-Arctic birch budburst, causing widespread tree die-off (50). Notably, interspecific variation in phenological responses may preserve ecosystem processes when species are not tightly linked, or when voltinism is plastic (51). The study of predator–prey mismatch remains challenging (52) and requires tracking how climate change is affecting organismal phenology at a high taxonomic and temporal resolution.

Our experiment is one of the few assessing phenological change at the community level in a realistic, outdoor mesocosm system (2); however, our approach has limitations. First, a multi-year experiment may have found greater declines in sensitive taxa abundance that are caused by inter-generational effects. High temperatures can reduce egg survival and adult fecundity via reduced body size, which may not be noticeable over a single season (27). Second, unmeasured abiotic variables beyond flow and temperature may have partly driven biotic responses. For example, reduced flow can increase retention of allochthonous particulate organic matter, which could influence biofilm metabolism (23); similarly, reduced water velocity may have influenced fluctuations in dissolved oxygen, even if biologically harmful hypoxia was not reached in our case [*SI Appendix,* Fig. S6 (53, 54)]. Reduced flow can also increase the concentration of nutrients with higher residence time, even if this is unlikely in our oligotrophic system (44, 55). Overall, disentangling abiotic change driven by vs. covarying with flow alteration requires further research. Third, we did not measure immigration or drift of individuals. While immigration from nearby Convict Creek during the experiment could have reduced treatment differences, drift into the channels was negligible in a past study (56). However, emigrant drift may have differed among our treatments, as described by studies that found short-term increases in drift under reduced flow conditions followed by drift declines (20). Some of our results could also be driven by a reduced period of high flow rather than by an earlier, longer summer low flow period, if organisms require prolonged high flow conditions for dispersal, filter-feeding, or some other life history aspect. Last, the artificial channels are not connected to groundwater, which can cool down low-order stream habitats experiencing summer low flow conditions (57). These factors suggest that care should be applied when transferring our results to other climatic and geologic contexts.

The temporal shifts in phenology and ecosystem processes we observed are meaningful given ongoing climate change trends in mountain ranges globally (19) and particularly in the rain-to-snow transition zone. In addition to climate change leading to advanced and extended summer low flow conditions, warmer air temperatures will increasingly overlap with periods of reduced thermal buffering from low flows (20, 58), increasing stream water temperatures even further (58). Warmer air temperatures will also increase the likelihood of precipitation falling as rain and rain-on-snow events, raising the frequency and magnitude of flooding (59). These changes in snow-dominated mountain streams are expected to cause widespread ecological change, as is already seen when comparing communities from unusually wet to dry years (15). Our study shows that response diversity may help maintain stability in key ecosystem processes, similarly to how biodiversity stabilizes ecosystem processes in warming terrestrial ecosystems [e.g., as seen with bee diversity and plant pollination (60)]. However, stabilizing mechanisms may be further eroded if environmental change continues to extirpate species locally (11). Studying community phenology at fine temporal scales is vital to capture the vulnerability of taxa facing climate change and to understand impending effects of climate change on ecosystem processes.

## Materials and Methods

### Experimental Design.

The experiment took place over 4 mo, from May 2019 to August 2019, in nine outdoor, flow-through channels at the Sierra Nevada Aquatic Research Lab (SNARL) located near Mammoth Lakes, California (*SI Appendix,* Fig. S1). The channels are 50 m long by 1 m wide, consist of six pools connected by long riffle sections in a meandering fashion, and are fed by the adjacent Convict Creek. Convict Creek also provided natural substrate consisting of cobbles, sand, and silt. This experimental array has been used in past research questions investigating fish growth and stream invertebrate community composition (56, 61). The artificial channels have the advantage of mimicking natural ecosystems better than recirculating field mesocosms or laboratory flumes, while allowing for replication that is difficult to obtain in natural streams. The channels were naturally colonized without alteration for over a year prior to the start of the experiment. We assigned each channel to one of three treatments (with three replicate channels each) in a block design. The three treatments were: 1) current hydrologic conditions based on the historic (long-term) hydrograph at Convict Creek (*SI Appendix,* Fig. S2), with a flow regime that reaches low flow conditions around August 3rd (i.e., *Current* treatment); 2) hydrologic conditions under a mitigated climate change scenario, where the stream would return to low flow conditions 3 wk earlier than it currently does (i.e., 3-wk treatment); and 3) hydrologic conditions under unmitigated climate change, where the stream would return to low flow 6 wk earlier than it currently does (i.e., 6-wk treatment). These scenarios connect greenhouse gas emission trajectories to the timing and duration of summer low flow (i.e., flow at or near designed low flow), based on a recent report using hybrid downscaling to project end-of-century hydrologic change in the Sierra Nevada (18).

We regulated discharge by controlling sluice gates at the head of each channel. Flows in the channels differed by one order of magnitude between high-flow and low-flow conditions (i.e., 15 L/s and 1.5 L/s, respectively), following a typical Sierra Nevada stream hydrograph for a small stream (*SI Appendix,* Fig. S3) (62). The 10-fold magnitude change in discharge, characteristic of Sierra Nevada streams, is due to the strong influence of snowmelt on the flow regime. We removed fish in the channels prior to the experiment, kept screens in place to exclude them (mesh size = 1.25 cm), and conducted electrofishing during the experiment to ensure their absence. These efforts allowed us to avoid confounding top–down effects and increase realism given first-order streams in the region tend to be fishless (unless artificially stocked). Channels were inspected and maintained daily, were heavily instrumented (see next section), and were monitored and sampled for several responses: epilithic biofilm metabolism, secondary production, and benthic and emerging stream invertebrates (composition and abundance). We tested whether each variable was explained by low flow treatment or, for time-varying variables, by period–treatment (i.e., the combination of time period and low-flow treatment). The three periods we designated in the study are: start (5/11/2019 to 6/10/2019), middle (6/11/2019 to 8/2/2019), and end (8/3/2019 to 8/21/2019). Period timespans were based on treatment timing: The start and middle periods are separated by the onset of summer low flow in the 6-wk treatment, and the middle and end periods are separated by the onset of summer low flow in the Current treatment. The number of sampling events was balanced among periods for biofilm production and benthic macroinvertebrates. However, more samples were taken in the middle period for emergent macroinvertebrates compared to the other periods, as a function of the higher frequency at which this ecological response was measured (i.e., every 10 d instead of 21), to account for its pulsated nature (63).

### Monitoring of Environmental Variables.

We measured water depth and water temperature every 5 min throughout the experiment (4/21/2019 to 8/25/2019) with replicated pressure transducers (HOBO U20L-04, Onset). We placed a pressure transducer in the fifth pool downstream in each channel and two emerged sensors on land to correct data for fluctuations in atmospheric pressure, and thus calculate water level (i.e., pool depth). Water level series were subsequently transformed into discharge series via channel-specific rating curves. Rating curves were developed for each channel by estimating discharge manually using channel depth and velocity measurements taken with a Marsh-McBirney Flo-Mate 2000 current meter throughout the summer (17 to 26 repeated estimates per channel). We measured water temperature using the same HOBO U20L-04 sensors that recorded data every 5 min in pools. We averaged discharge and water temperature to hourly values, which we then used to calculate daily metrics (i.e., daily mean, minimum, maximum, and diel range).

### Estimation of Epilithic Biofilm Metabolism.

We estimated epilithic biofilm production and respiration using the light/dark bottle method at each channel, once every 3 wk [as done previously (64)]. We calculated respiration (ER), net primary production (NPP), and the sum of their absolute values–gross primary production (GPP). We used three representative cobbles from the streambed for each sample and measured their surface area using aluminum foil to correct for differences in surface area. All epilithic biofilm production measurements were taken during peak sunlight hours between 10 AM and 2 PM using two 90-min incubation periods for light, followed by dark, measurements. Benthic stream invertebrates were removed from rocks prior to incubation. We conducted three replicates for each channel at each sampling date (*n* = 162). Daily GPP per channel was estimated by multiplying the channel average hourly rate by the number of sunlight hours at each date (*n* = 54). We estimated daily ER per channel by multiplying the channel average hourly rate by 24 h at each date (*n* = 54). Daily epilithic biofilm production was then estimated for the interval between each sampling date by averaging the bookend interval values. We multiplied the average interval value by the number of days in the interval and finally summed these values to generate cumulative seasonal channel estimates (*n* = 9). GPP and ER can both be higher during the day when sunlight and temperatures peak, so there is some uncertainty around our extrapolated daily and seasonal estimates. We also collected continuous dissolved oxygen series in all channels and in the feeder channel, but short water residence times in the channels prevented us from using diel variation in dissolved oxygen to model whole-ecosystem metabolism.

### Sampling and Processing Benthic Invertebrates.

We sampled benthic stream invertebrates using a 500-micron Surber sampler at six visit dates 3 wk apart throughout the experiment. Each sample was a composite of three subsamples (two riffle and one pool samples for 0.279 m^2^ total) to represent the overall stream community. We took benthic samples for the Current and 6-wk treatment channels (*n* = 36) and stored them in 70% ethanol. We then subsampled the composite samples using a rotating-drum splitter in the laboratory to sort and identify at least 500 individuals from each composite sample under a stereomicroscope. All subsamples were completely processed to avoid bias regarding the size of individuals picked and identified. Benthic stream invertebrates were identified to the highest resolution possible, typically genus or species level, and all intact specimens were measured. Benthic stream invertebrate biomass was then estimated using published taxon-specific length-mass relationships (65–69). The subsampled community was multiplied by the inverse of the fraction of the total sample that was identified (e.g., if ¼ of the sample was identified to get a count over 500 individuals, then the abundance of each taxon was multiplied by 4). We assigned length values to these extrapolated individuals (and individuals that could be identified but not measured due to damage) using the length values from randomly selected individuals of the same taxon in the sample.

We sampled emergent stream invertebrates using emergence traps, each deployed for 72 h every 3 wk during the experiment. We sampled emergence four additional times halfway between the 3-wk intervals for every sample visit after the second one, when flows began to differ between treatments (*n* = 90 overall). We deployed emergence traps at the tail of riffles (to capture the influence of both riffle and pool habitat) next to HOBO sensors. We identified emergent insects to genus or family level (depending on taxa), and measured length of intact specimens. Emergence traps were tent-shaped, covered 0.33 m^2^ of the stream, and had 0.2-mm white mesh (70). We chose to use emergence traps over sticky traps or other alternatives because they do not damage individuals, allowing for fine taxonomic identification that is critical to assess phenology (71). We derived seasonally aggregated values of benthic and emergent abundance or flux, respectively, as the sum of all samples taken for each channel.

### Secondary Production.

We estimated benthic stream invertebrate secondary production via a combination of three methods. We used the size-frequency method for taxa that were abundant throughout the experiment (i.e., >1% of total abundance) and had known generation times, excluding Chironomidae, Oligochaeta, Turbellaria, and Muscidae (72). For Chironomids, we used the instantaneous growth rate method. Production was calculated using regression equations for non-Tanypodinae chironomids, which incorporate mean temperature into growth estimates for small, medium, and large chironomids (73). Finally, we used the production to biomass ratio method (P/B) for the remaining taxa, including Tanypodinae, by multiplying seasonal biomass by known P/B ratios in the literature of the closest related taxa possible (74, 75). Uncertainty in production from P/B ratios is unlikely to affect our results, as taxa in this group comprised <1% of the total assemblage production. We estimated emergent insect biomass using published, taxon-specific length-mass relationships (76). We derived seasonally aggregated estimates of emergent production by taxon, by multiplying the average biomass between successive samples by the number of days in the interval, and by then summing interval estimates for the season.

### Brewer’s Blackbird Feeding Observations.

We noticed Brewer's Blackbirds (*Euphagus cyanocephalus*) feeding in the 6-wk treatment channels at the onset of summer low flow (June 22, 2019). Brewer’s Blackbirds were nesting nearby and waded in the channels to pick benthic macroinvertebrates as food for their young. We recorded feeding behavior of Brewer’s Blackbirds shortly thereafter to examine whether they altered the invertebrate community in the channels. We studied Brewer’s Blackbird behavior by observing the time duration that any bird occupied the benthos of the channels over a 30-min period. We measured this behavior with a stopwatch and made observations periodically throughout the remainder of the experiment between noon and 6 PM (77). We switched our target from daily to weekly observations once Brewer’s Blackbirds fledged and moved to meadow habitat, far (>5 km) from the channels. Two researchers conducted these observations each time, with one person observing the six upper channels and another person observing the three lower channels. Brewer’s Blackbirds were not observed feeding in the channels before summer low flow, as the high water depth prevented them from wading and they were not yet nesting at that point.

### Data Analysis.

For our first prediction that extended summer low flow would shift epilithic biofilm metabolism phenology, we tested GPP:ER, GPP, and ER across period–treatment using repeated measures ANOVA and pairwise post hoc comparisons with the Benjamini–Hochberg correction when appropriate (i.e., when period–treatment was significant). We log transformed GPP:ER to improve the normality of residuals. We assessed cumulative season-long GPP:ER across treatments using a two-way ANOVA in order to assess whether epilithic biofilm metabolism varied across low flow treatments. GPP and ER were tested similarly. Several ANOVA and repeated measures ANOVA tests throughout our analyses violated the assumption of equal variance (based on a Fligner–Killeen test) but were still the best available method to test our questions. In such cases, we visually confirmed that statistical patterns were not driven by a single sample with high leverage.

For our second prediction regarding stream invertebrate phenology and production, we first used permutational multivariate ANOVA (PERMANOVA) tests based on 999 permutations with the function *adonis2* in the *vegan* R package in order to quantify benthic and emergent community change over time and across treatments (78). We also ran pairwise post hoc comparisons with the Benjamini–Hochberg correction, when appropriate. We estimated community dissimilarity using the Bray–Curtis statistic, and visualized community trajectories via non-metric multidimensional scaling (NMDS). We fit individual taxa using the function *envfit*, also in the *vegan* package, and subsequently filtered the taxa based on which had a highly significant correlation with the NMDS axes (*P* ≤ 0.002; *SI Appendix*, Fig. S10 and Tables S6 and S12). Taxa that were significantly correlated with a NMDS axis were further tested for variation in abundance across period–treatment.

In order to quantify how period–treatment may change benthic stream invertebrate taxa populations and emergent flux in aquatic insects, we used repeated measures ANOVA tests and pairwise post hoc comparisons with the Benjamini–Hochberg correction when appropriate. We square-root transformed taxa abundance when needed to improve normality of residuals, although some skewed distributions did not strictly pass the homogeneity of variance test. We tested whether shifts in scraper abundance (i.e., grazing invertebrates) occurred across period–treatment levels to examine the possibility that the experimental treatment altered top–down (herbivory) control. To this end, we assigned taxa to functional feeding groups (using ref. 79) and pooled all scrapers to assess their change over time and treatments.

In order to test whether cumulative seasonal benthic stream invertebrate secondary production responded to low flow treatment, we used 95% CIs of bootstrapped data (*n* = 1,000) from each channel (72). The 95% CIs of the treatments included the 97.5th and the 2.5th percentiles of all values from the same treatment. We tested whether low flow treatments affected cumulative seasonal emergent production for the community and individual emergent taxa using a two-way ANOVA.

Last, we tested response diversity of the 15 most abundant benthic macroinvertebrates using dissimilarity abundance responses to discharge change. We first took the derivative of the relationship between abundance and discharge for each species, then estimated dissimilarity based on pairwise Euclidean distances in derivatives between all pairs of species in the community (following ref. 36). We excluded our first sampling date so that low discharge always corresponded with warmer conditions. We compared the distribution of dissimilarity values in our study with previous values reported in the literature, as benchmarks of low and high response diversity (dissimilarity) levels (36).

For our third prediction, we used piecewise structural equation models with the *psem* function in the *piecewiseSEM* R package in order to mechanistically test the relationships between discharge, water temperature, benthic production, and emergent production (80). Piecewise structural equation models allow more flexibility in model structure (which we needed to run repeated measures linear mixed effect models) than traditional structural equation models. We focused on emergent Chironomidae, as that was the only taxon that had time-varying benthic production (i.e., the instantaneous growth rate method provided time-varying secondary production, unlike other methods). Calculated growth rates for taxa are rare in the literature and were unavailable for other taxa in the study. We calculated benthic production using the average biomass between sampling dates, so that five sampling midpoints were used for analysis in the model. All other variables were averaged for sample midpoint dates matching benthic production (*n* = 30). We further tested the mechanisms and links between discharge and Chironomidae production with a second model that used benthic and emergent size along with benthic and emergent abundance. All six sampling dates were used in this case (*n* = 36). We log transformed benthic abundance, emergent abundance, and emergent production to give model residuals a normal distribution. Both piecewise structural equation models were supported for inference based on the Fisher’s *C* statistic. Model coefficients were standardized by SD for comparison.

We also used repeated measures ANOVA tests in order to analyze how period–treatment affected emergent Chironomidae production. We used pairwise post hoc comparisons with the Benjamini–Hochberg correction for the ANOVA data. Last, we tested Brewer’s Blackbird feeding time using the Kruskal–Wallis rank sum test. We specifically tested whether the time that Brewer’s Blackbirds were observed in the channel was explained by low flow treatment. We also tested whether Brewer’s Blackbirds altered the macroinvertebrate community following a before-after-control-impact (BACI) design, focusing on the interaction term between treatment and time (i.e., before vs. after blackbird presence). We ran a total of six different tests, to explore potential effects of blackbirds on benthic and emergent invertebrate richness and abundance (via repeated measures ANOVA models), and on benthic and emergent composition (via a PERMANOVA, given the multivariate nature of the data). If Brewer’s Blackbirds caused an effect, we would expect the interaction term to be significant, reflecting a “difference in difference” between the 6-wk and Current treatment after the blackbirds’ arrival.

## Supplementary Material

Appendix 01 (PDF)

## Data Availability

Data and code data have been deposited in Dryad (81).
