# Supplementary material for: Climate change is poised to alter mountain stream ecosystem processes via organismal phenological shifts
Source: Proc Natl Acad Sci U S A. 2024 Mar 18;121(14):e2310513121. doi: 10.1073/pnas.2310513121 (PMC10998557; doi:10.1073/pnas.2310513121)
Supplement: Supplementary file 1 — Appendix 01 (PDF) [file pnas.2310513121.sapp.pdf]

PNAS

Supporting Information for:

Climate change is poised to alter mountain stream ecosystem processes via organismal phenological shifts

Kyle Leathers[1*], *David Herbst*[2], *Guillermo de Mendoza*[1,3], *Gabriella Doerschlag*[1], *Albert Ruhi*[1]

[1] Department of Environmental Science, Policy, and Management, University of California, Berkeley, Berkeley, CA 94720, USA

[2] Sierra Nevada Aquatic Research Laboratory, University of California, 1016 Mt. Morrison Road, Mammoth Lakes, CA 93546, USA

[3] Institute of Biology and Earth Sciences, Pomeranian University in Słupsk, Słupsk, 76-200, Poland

*Correspondence should be addressed to Kyle Leathers

Email: kyle_leathers@berkeley.edu

This PDF file includes:

Figures S1 to S10
Tables S1 to S15
SI References

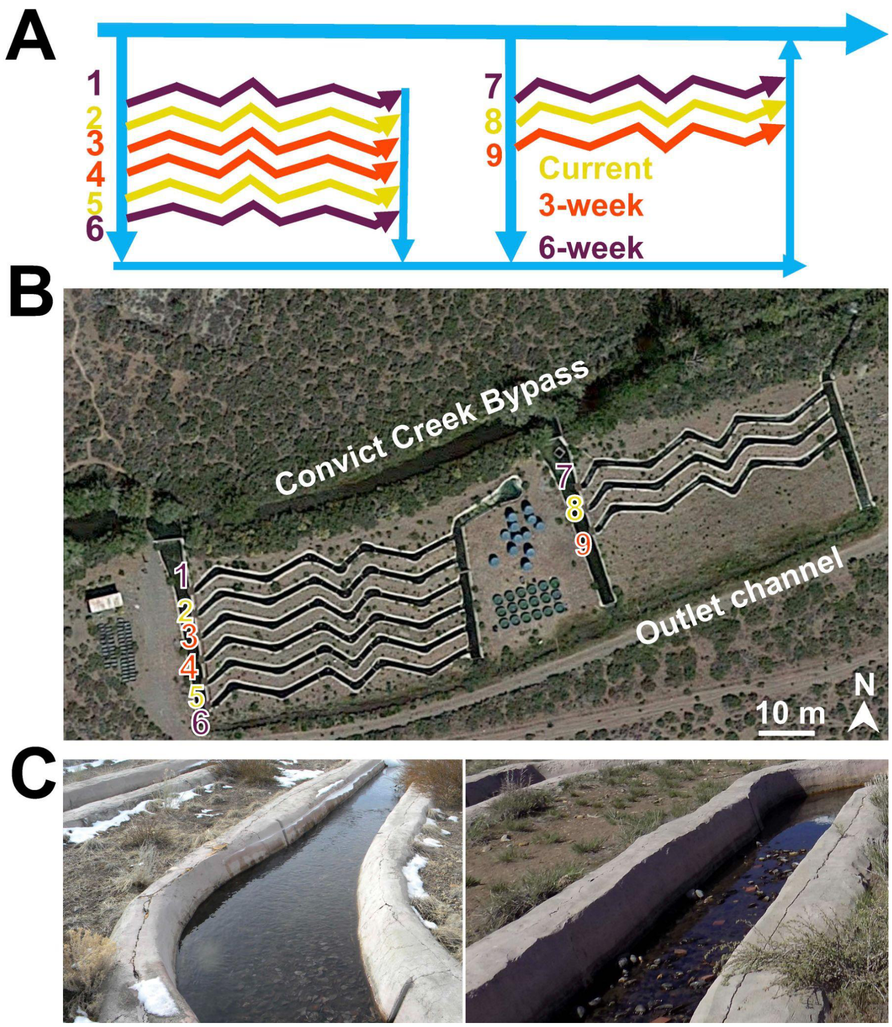

Figure S1. Diagram and photos of the outdoor channel array at the Sierra Nevada Aquatic Research Laboratory (SNARL) in Mammoth Lakes, California, USA. (**A**) Channels were assigned to one of three treatments with three replicate channels each in a block design. Channel number is shown to the left of the channel inlet and colored according to treatment. The treatments were: (1) current hydrologic conditions based on the historic (long-term) hydrograph at Convict Creek with a flow regime that reaches baseflow conditions around August 3rd (i.e., *Current* treatment); (2) hydrologic conditions under a mitigated climate change scenario, where the stream would return to baseflow conditions three weeks earlier than it currently does (i.e., *3-week* treatment); and (3) hydrologic conditions under unmitigated climate change, where the stream would return to baseflow six weeks earlier than it currently does (i.e., *6-week* treatment). (**B**) Aerial photo of the artificial channels, courtesy of Google Maps. (**C**) Photos of high flow (left; ~15 L/s) and low flow (i.e., baseflow) conditions (right; ~1.5 L/s). Photo credits: Carol Blanchette (B), Guillermo de Mendoza (C1), and Ludmila Sromek (C2).

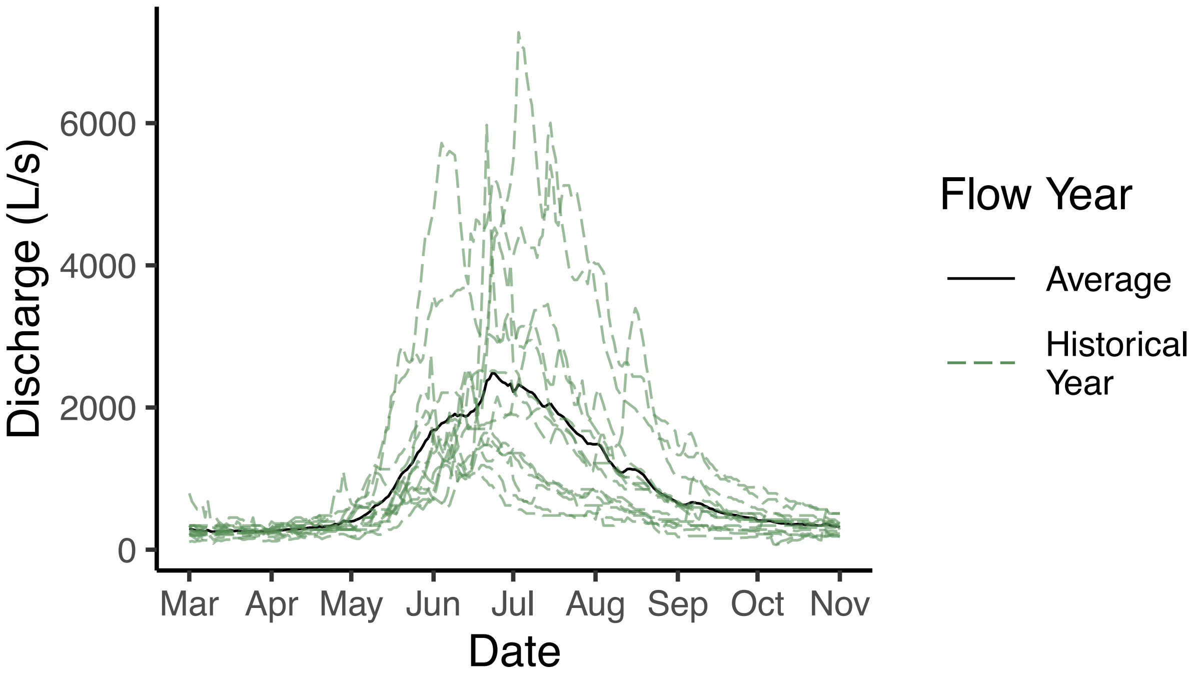

Figure S2. Historical flow regime of Convict Creek. Long-term seasonal trends in mean daily discharge in Convict Creek from March to November each year from 1960 to 1974 (in dashed green lines), as measured by the available data from U.S. Geological Survey streamgage 10265200. We averaged the 15 years of streamflow data to create a daily mean discharge value (black line). We then used this average flow regime as the basis for the "*Current*" flow regime and advanced the start of summer low flows for the *3-week* and *6-week* treatments from this baseline, in agreement with downscaled climate change projections from Reich et al. 2018 (1; Figure S3). The first-order streams that our experimental channels are simulating are smaller than Convict Creek and located at a higher altitude. They thus experience faster return to summer baseflow conditions.

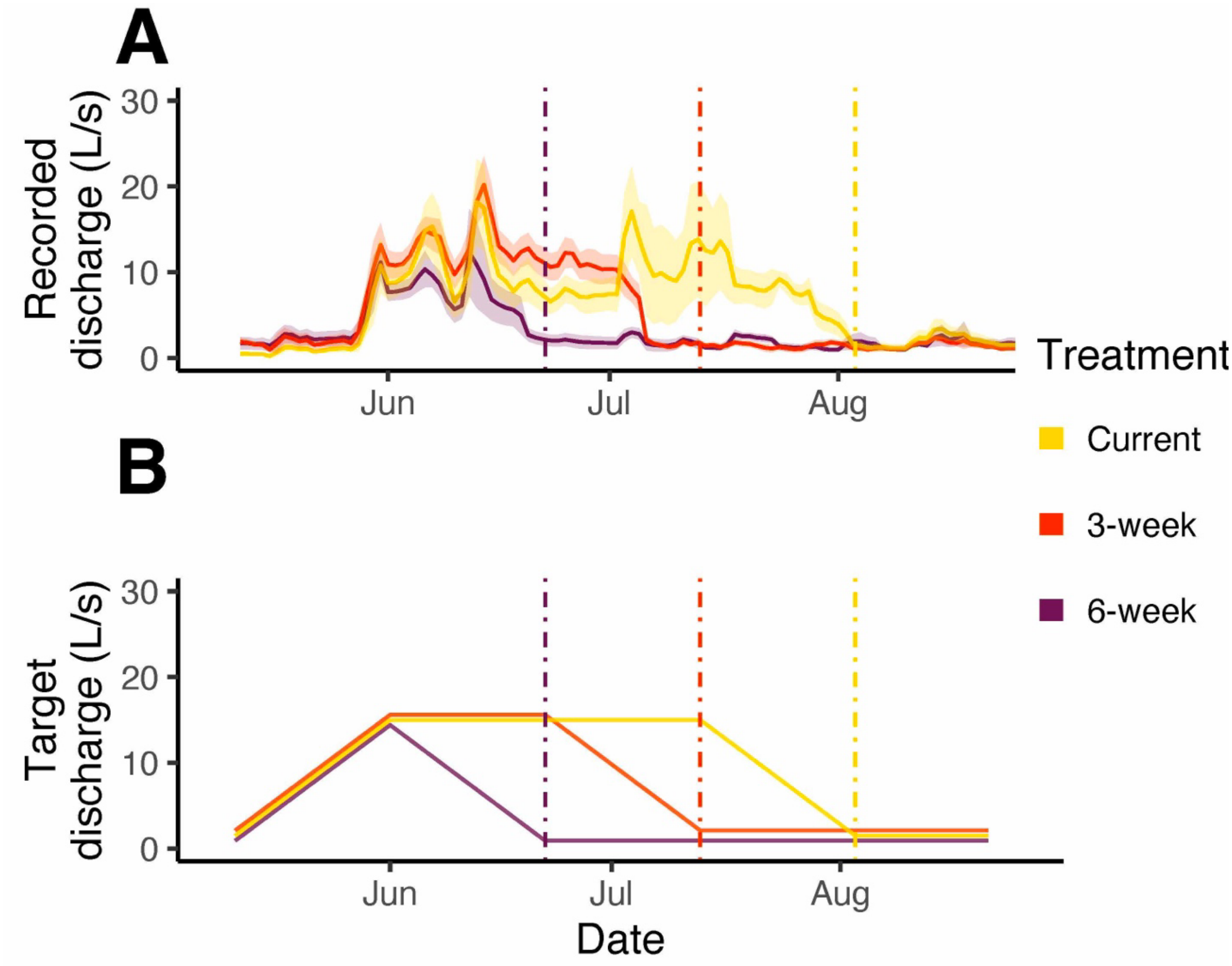

Figure S3. Average recorded and targeted daily discharge for each treatment throughout the experiment. (**A**) The recorded discharge was calculated using high frequency water depth sensors and rating curves in each channel. Discharge was manually controlled by opening or closing the sluice gate at the inlet of each channel to match the target flow regime. (**B**) Target discharge in the *Current* treatment follows the historic average flow regime timing of snowmelt recession in the water source, Convict Creek (see Fig. S3). The other two treatments were designed to experience summer low flow three and six weeks earlier in the year, respectively, following downscaled climate change projections (see methods for details). The designed onset of summer low-flow conditions is indicated for each treatment by a dashed vertical line colored by treatment. The shaded area represents ±1 standard error.

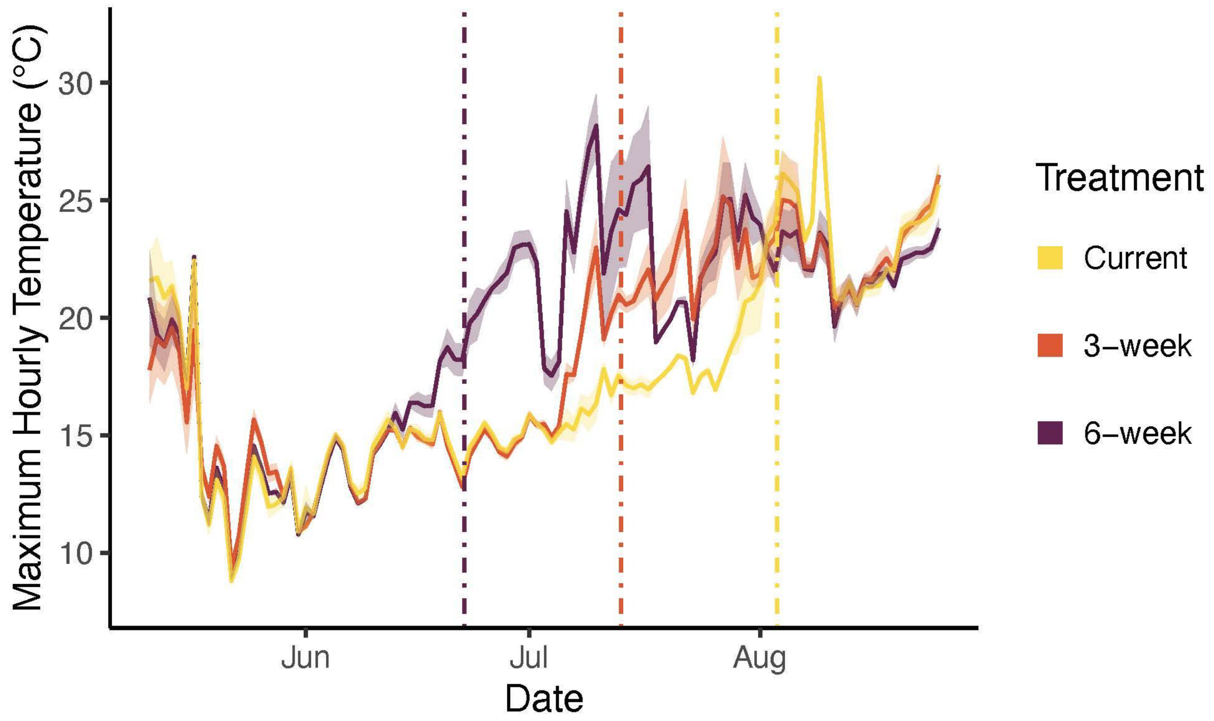

Figure S4. Maximum daily water temperature for each treatment, throughout the experiment. Maximum water temperature increased with advanced low flow treatments. The targeted onset of summer low-flow conditions is indicated for each treatment by a dashed vertical line, colored by treatment. Average treatment maximum water temperature (± standard error) is shown. See Figure S3 for targeted and observed hydrographs.

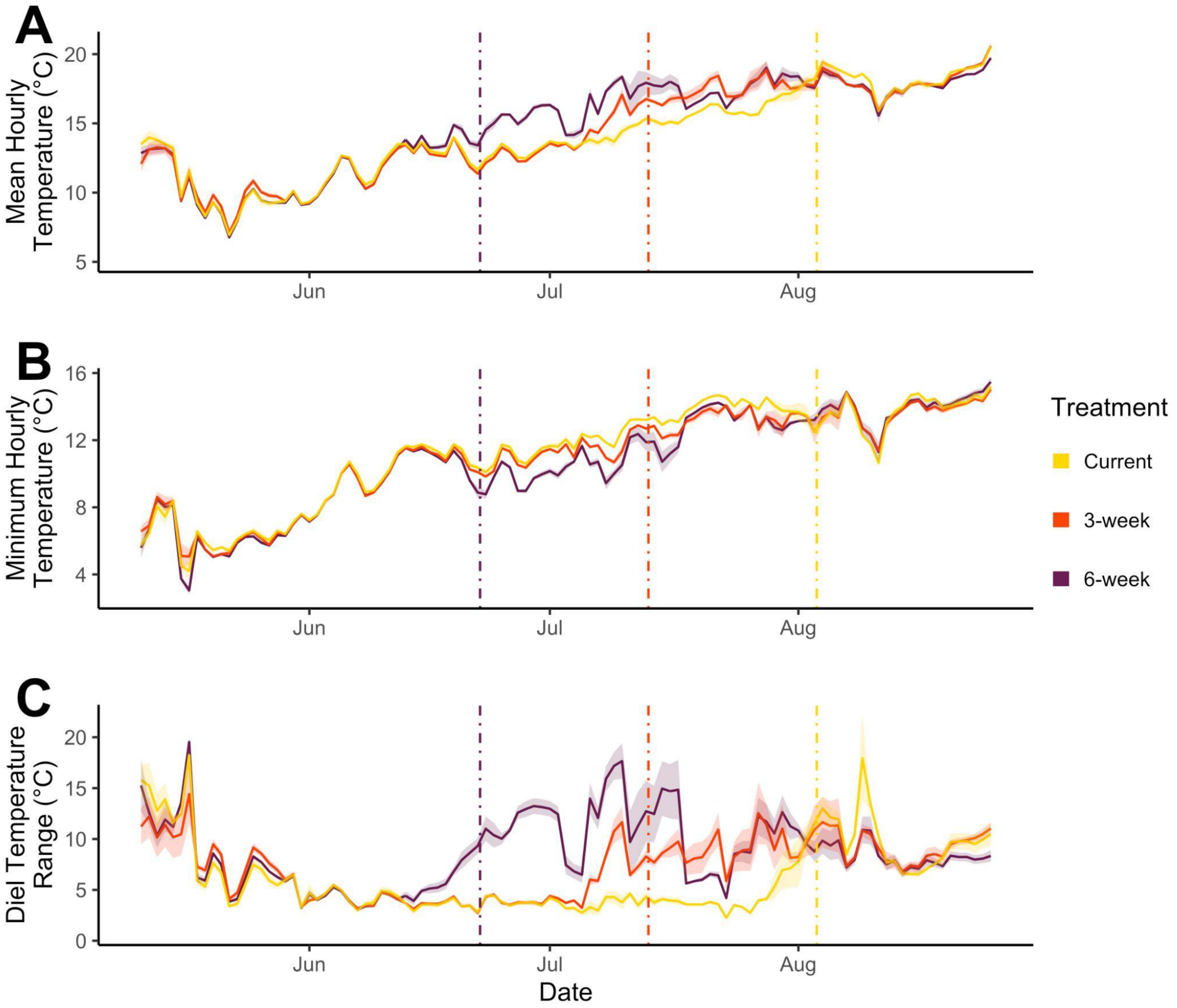

Figure S5. Mean, minimum, and diel range of water temperature for each treatment throughout the experiment. Advanced low flow treatments affected all water temperature metrics immediately. The designed onset of summer low-flow conditions is indicated for each treatment by a dashed vertical line colored by treatment. The means of each water temperature metric (± standard error) are shown by treatment.

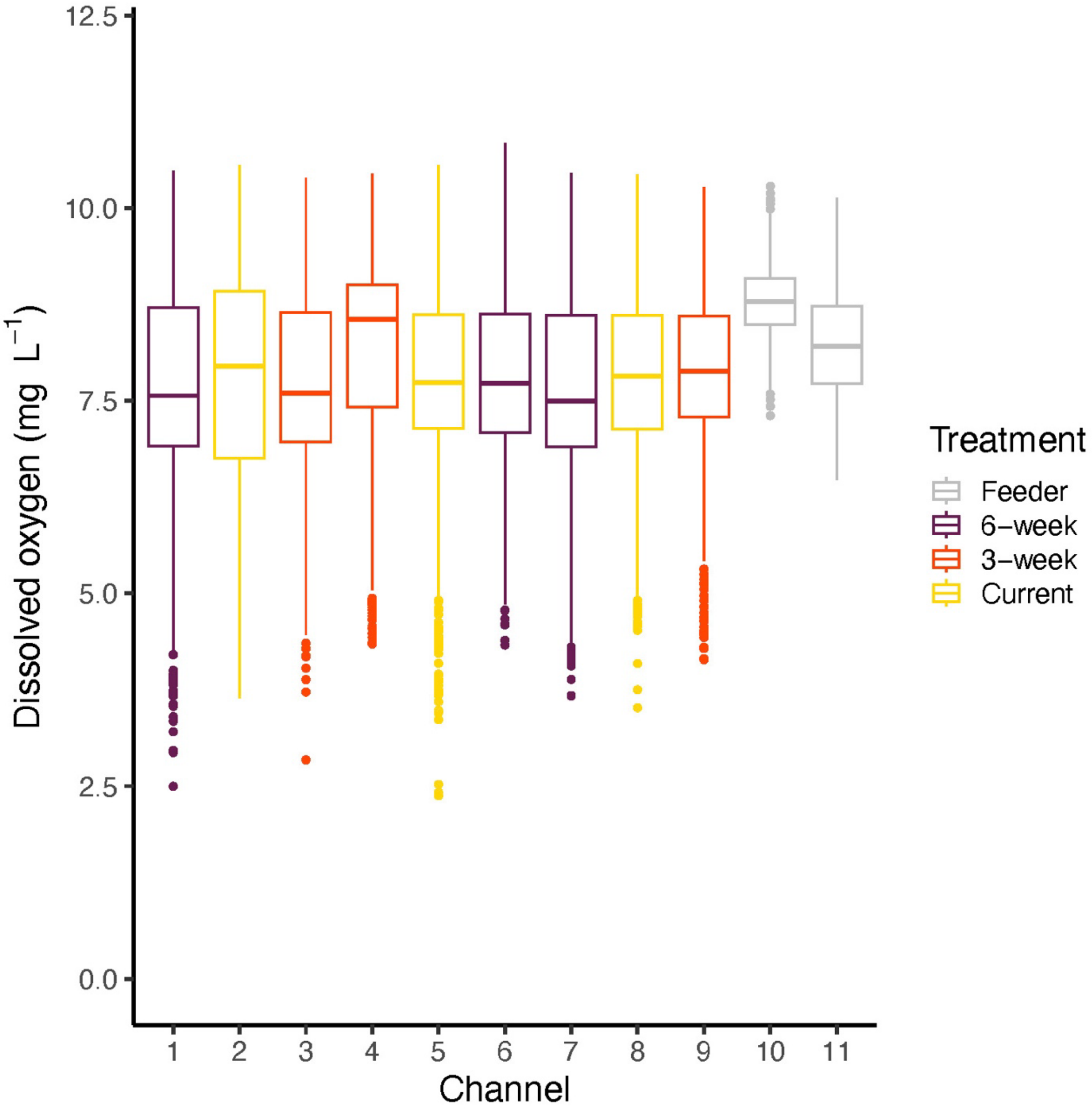

Figure S6. Distribution of dissolved oxygen hourly concentrations in each channel, pooled across the duration of the experiment. The boxplots display channel-specific medians (bold lines), interquartile ranges (boxes), and interquartile range*1.5 extents (whiskers). Hypoxia, defined as dissolved oxygen concentrations dropping below 2 mg/L (2), was never detected in the experiment, and almost all readings (99.6% of them) were over 5 mg/L, a threshold often used to assess long-term performance of sensitive, coldwater mayflies and stoneflies (3 and references therein). The "Feeder" label refers to measurements taken at the two inlets connecting Convict Creek to the experimental channels.

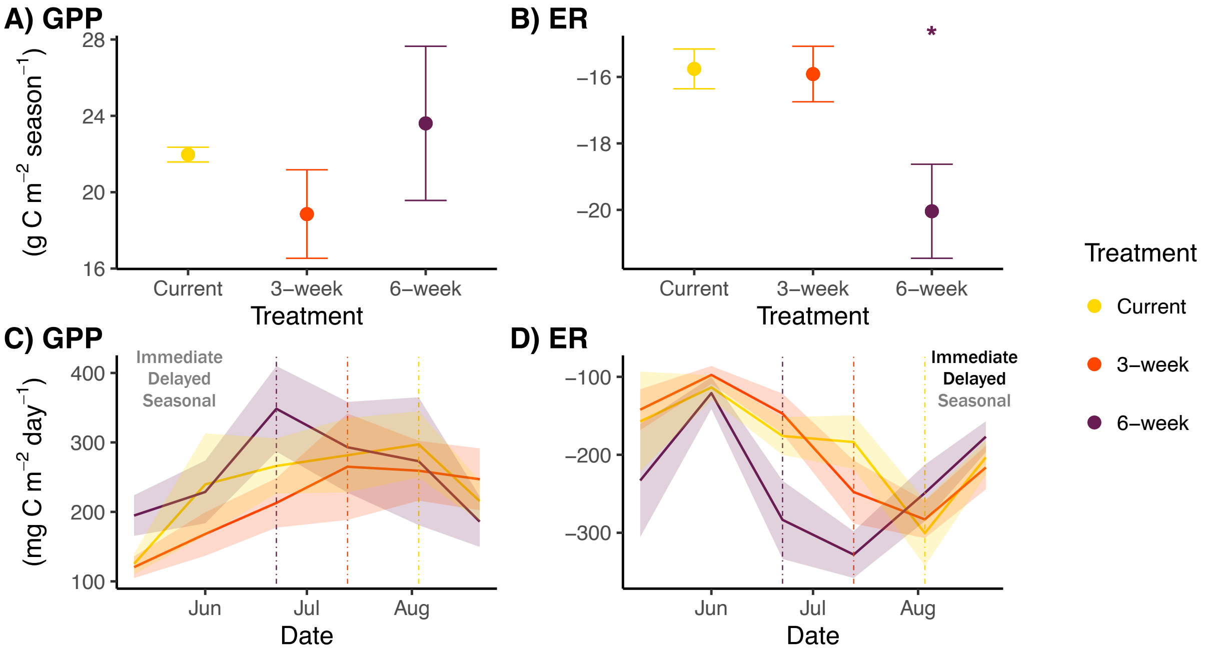

Figure S7. Seasonal and time-varying estimates of epilithic biofilm production (GPP) and respiration (ER). (**A**) No differences were observed between treatments for cumulative seasonal GPP. (**B**) Cumulative seasonal ER was significantly higher in magnitude for the *6-week* treatment relative to the other two treatments (indicated by *). (**C**) No predicted responses were observed for GPP. (**D**) An immediate increase in the magnitude of ER occurred in the *6-week* treatment based on comparisons between the *middle* and *start* of the experiment. Shaded error ribbons and error bars represent ±1 standard error. Each breakpoint in the time series plots denotes a sampling event. The three potential response types (*immediate* treatment effect, *delayed* treatment effect, and *seasonal* effect) are listed, and colored in black when supported (see conceptual framework in Fig. 1, and Table S1 for how statistical tests connect with each response type).

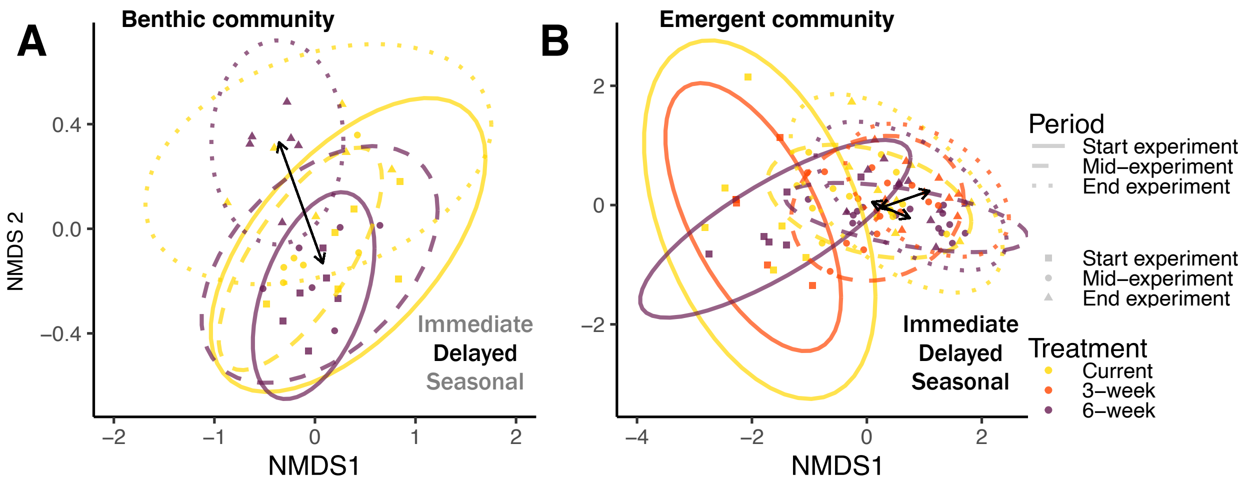

Figure S8. Low-flow treatment effects on stream invertebrate community structure. Earlier, extended low flows had a delayed effect on benthic stream invertebrate community composition and an immediate effect on emergent insect community composition. Black arrows represent significant post-hoc differences due to predicted responses, where arrow end points indicate ellipse centroids. (**A**) The benthic stream invertebrate community experienced a *delayed* response, based on differences between the *6-week* treatment at the *end* and the *middle* of the experiment. (**B**) The community composition of emergent stream invertebrates changed immediately in the *middle* period between the *6-week* treatment and the *Current* treatment. There was a *delayed* change in the community in the *3-week* treatment. The *middle* period in this study begins ten days preceding the onset of summer low flow for the *6-week* treatment, to reflect that falling discharge can cause effects before minimum summer low flow is reached. The three potential response types (*immediate* treatment effect, *delayed* treatment effect, *seasonal* effect) are listed, and colored black when supported (see conceptual framework in Fig. 1, and Table S1 for how statistical tests connect with each response type).

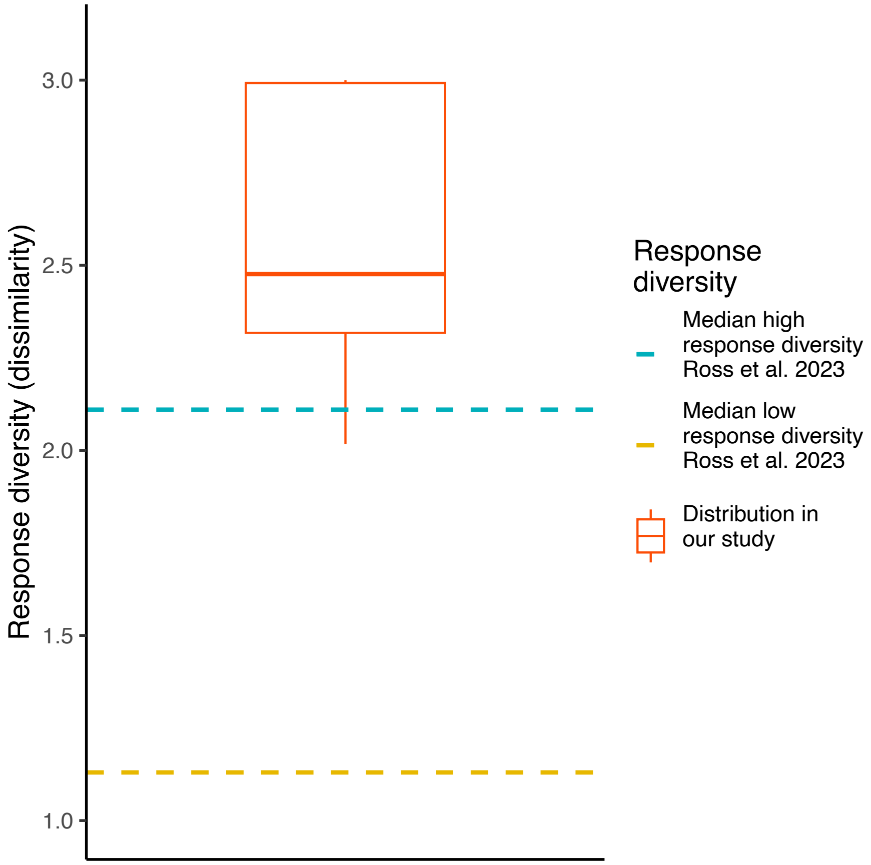

Figure S9. Response diversity in our study relative to values in the literature. We measured response diversity using dissimilarity in how species abundance responded to change in discharge, via the method described in Ross et al. 2023 (4). We note dissimilarity has a minimum value of 1, and higher values indicate higher response diversity. The 15 most abundant benthic taxa were included in our analyses, and response diversity was calculated for each channel independently. The boxplot displays the median (bold red line), interquartile range (box), and interquartile range*1.5 (whiskers) of all channel response diversity values. Samples from the first sampling date were excluded for this analysis to focus on the experimental response. The dashed horizontal lines are median dissimilarity values used as benchmarks, reported to have low and high dissimilarity in Ross et al. 2023.

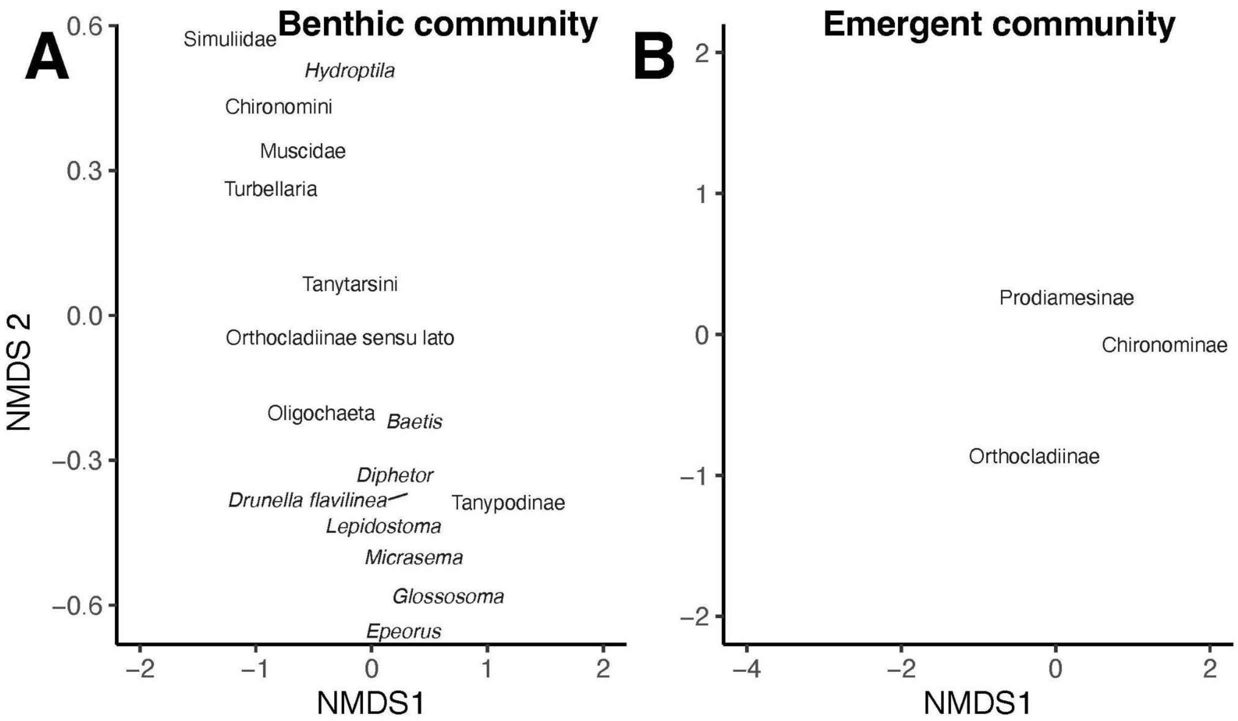

Figure S10. Taxa driving community dissimilarity in Figure S8. The NMDS space is the same here as that in Figure S8. (**A**) Benthic taxa that significantly explained community dissimilarity among samples are shown ($P \leq 0.002$). The position of the taxa label indicates where the taxa is in the NMDS space. The label position for *Drunella flavilinea* is moved to prevent overlap and the line connected to it indicates the actual space it corresponds to in the NMDS. (**B**) Emergent taxa that significantly ($P \leq 0.002$) explained community dissimilarity among samples.

Table S1. Description of how statistical tests support evidence for each of the potential ecological responses described in Figure 1 (*no* effect, *seasonal* effect, *immediate* treatment effect, *delayed* treatment effect). Responses reflect a change in magnitude or phenology of the response variable. A *seasonal* effect is inferred when an advanced low flow treatment and the *Current* conditions treatment exhibit a shift in the same direction and magnitude over time. An example of a *seasonal* effect is Chironominae emergence (Figure 3; Table S13), where all three treatments increase in emergence in the *middle* period compared to the *start* period. An *immediate* effect occurs when there is a difference in how treatments change from the *start* to the *middle* period, or when a difference occurs between treatments within the *middle* period (i.e., at the onset of treatment differences). An *immediate* effect is seen in Chironominae, where emergence under the *6-week* treatment is significantly different from emergence under the *Current* treatment (*middle* period). Lastly, a *delayed* effect occurs when there is a difference in how treatments change from the *middle* to *end* period, or between treatments within the *end* period. An example of a *delayed* effect occurred in benthic Chironomini (Figure 3), where abundance increased significantly between the *middle* and *end* periods for the *6-week* treatment, but not for the *Current* treatment. Immediate and delayed effects could theoretically occur sequentially in the same response variable, but we did not expect it a priori, and it did not occur in our study.

**Response type**	**Relevant comparisons**	**Rationale**
Seasonal effect	Middle 6-week *vs.* Start 6-week<br>Middle Current *vs.* Start Current<br>Middle 3-week *vs.* Start 3-week<br>End 6-week *vs.* Middle 6-week<br>End Current *vs.* Middle Current<br>End 3-week *vs.* Middle 3-week	If the C*urrent* treatment and either the *6-week* or *3-week* treatment exhibit the same significant response over time, then we describe a seasonal effect.
Immediate effect	Middle 6-week *vs.* Start 6-week<br>Middle Current *vs.* Start Current<br>Middle 3-week *vs.* Start 3-week<br>Middle 6-week *vs.* Middle Current<br>Middle 3-week *vs.* Middle Current	An immediate effect may be evidenced by a significant difference between the C*urrent* and *6-week* or *3-week* treatments in the *middle* period. An immediate effect can also take place if the C*urrent* and *6-week* or *3-week* treatments differ in how they change between the *start* and *middle* periods.
Delayed effect	End 6-week *vs.* Middle 6-week<br>End Current *vs.* Middle Current<br>End 3-week *vs.* Middle 3-week<br>End 6-week *vs.* End Current<br>End 3-week *vs.* End Current	A delayed effect may be evidenced by a significant difference between the C*urrent* and *6-week* or *3-week* treatments in the *end* period. A delayed effect can also take place if the C*urrent* and *6-week* or *3-week* treatments differ in how they change between the *middle* and *end* periods.

Table S2. Pairwise comparisons of maximum water temperature among *period-treatment* groups. We examined maximum daily temperature via post-hoc pairwise comparisons (*P*-values) corrected by the Benjamini–Hochberg method. A bolded *P*-value indicates that the pairwise comparison was significant for that response type. If the *Current* and *3-week* or *6-week* treatments are significant for the same comparison across periods (e.g., *start* vs. *middle*) then *P*-values are bolded for *seasonal* effects but not for *immediate* or *delayed* treatment effects, as that would indicate similar change over time regardless of treatment. See conceptual framework on response types in Fig. 1, and Table S1 for how statistical tests connect with each response type.

**Response type**	**Relevant comparisons**	***P*-value**
Seasonal effect	Middle 6-week *vs.* Start 6-week	**1.07E-12**
	Middle Current *vs.* Start Current	**5.49E-06**
	Middle 3-week *vs.* Start 3-week	**1.81E-09**
	End 6-week *vs.* Middle 6-week	**0.0231**
	End Current *vs.* Middle Current	**3.93E-12**
	End 3-week *vs.* Middle 3-week	**2.02E-09**
Immediate treatment effect	Middle 6-week *vs.* Start 6-week	1.07E-12
	Middle Current *vs.* Start Current	5.49E-06
	Middle 3-week *vs.* Start 3-week	1.81E-09
	Middle 6-week *vs.* Middle Current	**1.35E-09**
	Middle 3-week *vs.* Middle Current	**1.85E-04**
Delayed treatment effect	End 6-week *vs.* Middle 6-week	0.0231
	End Current *vs.* Middle Current	3.93E-12
	End 3-week *vs.* Middle 3-week	2.02E-09
	End 6-week *vs.* End Current	**0.017**
	End 3-week *vs.* End Current	0.425

Table S3. Pairwise comparisons of dissolved oxygen among *period-treatment* groups. We examined dissolved oxygen over time via post-hoc pairwise comparisons (*P*-values) corrected by the Benjamini–Hochberg method. A bolded *P*-value indicates that the pairwise comparison was significant for that response type. If the *Current* and *3-week* or *6-week* treatments are significant for the same comparison across periods (e.g., *start* vs. *middle*) then *P*-values are bolded for *seasonal* effects but not for *immediate* or *delayed* treatment effects, as that would indicate similar change over time regardless of treatment. See conceptual framework on response types in Fig. 1, and Table S1 for how statistical tests connect with each response type.

Response type	Relevant comparisons	*P*-value
Seasonal effect	Middle 6-week *vs.* Start 6-week	**1.78E-06**
	Middle Current *vs.* Start Current	**2.16E-05**
	Middle 3-week *vs.* Start 3-week	**9.83E-06**
	End 6-week *vs.* Middle 6-week	0.918
	End Current *vs.* Middle Current	6.12E-03
	End 3-week *vs.* Middle 3-week	9.99E-02
Immediate treatment effect	Middle 6-week *vs.* Start 6-week	1.78E-06
	Middle Current *vs.* Start Current	2.16E-05
	Middle 3-week *vs.* Start 3-week	9.83E-06
	Middle 6-week *vs.* Middle Current	4.33E-01
	Middle 3-week *vs.* Middle Current	8.93E-01
Delayed treatment effect	End 6-week *vs.* Middle 6-week	0.918
	End Current *vs.* Middle Current	**6.12E-03**
	End 3-week *vs.* Middle 3-week	9.99E-02
	End 6-week *vs.* End Current	**0.042**
	End 3-week *vs.* End Current	0.133

Table S4. Pairwise comparisons of epilithic biofilm GPP:ER ratios among *period-treatment* groups. We examined GPP:ER ratios over time via post-hoc pairwise comparisons (*P*-values) corrected by the Benjamini–Hochberg method. A bolded *P*-value indicates the pairwise comparison was significant for that response type. If the *Current* and *3-week* or *6-week* treatments are significant for the same comparison across periods (e.g., *start* vs. *middle*), then *P*-values are bolded for *seasonal* effects but not for *immediate* or *delayed* treatment effects, as that would indicate similar change over time regardless of treatment. See conceptual framework on response types in Fig. 1, and Table S1 for how statistical tests connect with each response type.

**Response type**	**Relevant comparisons**	***P*-value**
Seasonal effect	Middle 6-week *vs.* Start 6-week	7.21E-02
	Middle Current *vs.* Start Current	9.89E-01
	Middle 3-week *vs.* Start 3-week	9.63E-01
	End 6-week *vs.* Middle 6-week	0.989
	End Current *vs.* Middle Current	1.12E-02
	End 3-week *vs.* Middle 3-week	2.26E-01
Immediate treatment effect	Middle 6-week *vs.* Start 6-week	7.21E-02
	Middle Current *vs.* Start Current	9.89E-01
	Middle 3-week *vs.* Start 3-week	9.63E-01
	Middle 6-week *vs.* Middle Current	**1.12E-02**
	Middle 3-week *vs.* Middle Current	1.71E-01
Delayed treatment effect	End 6-week *vs.* Middle 6-week	0.989
	End Current *vs.* Middle Current	**1.12E-02**
	End 3-week *vs.* Middle 3-week	2.26E-01
	End 6-week *vs.* End Current	0.937
	End 3-week *vs.* End Current	0.989

Table S5. Pairwise comparisons of biofilm respiration among *period-treatment* groups. We examined biofilm respiration over time via post-hoc pairwise comparisons (*P*-values) corrected by the Benjamini–Hochberg method. A bolded *P*-value indicates the pairwise comparison was significant for that response type. If the *Current* and *3-week* or *6-week* treatments are significant for the same comparison across periods (e.g., *start* vs. *middle*) then *P*-values are bolded for *seasonal* effects but not for *immediate* or *delayed* treatment effects, as that would indicate similar change over time regardless of treatment. See conceptual framework on response types in Fig. 1, and Table S1 for how statistical tests connect with each response type.

**Response type**	**Relevant comparisons**	***P*-value**
Seasonal effect	Middle 6-week *vs.* Start 6-week	3.55E-04
	Middle Current *vs.* Start Current	1.51E-01
	Middle 3-week *vs.* Start 3-week	3.07E-02
	End 6-week *vs.* Middle 6-week	0.0242
	End Current *vs.* Middle Current	4.86E-02
	End 3-week *vs.* Middle 3-week	1.78E-01
Immediate treatment effect	Middle 6-week *vs.* Start 6-week	**3.55E-04**
	Middle Current *vs.* Start Current	1.51E-01
	Middle 3-week *vs.* Start 3-week	**3.07E-02**
	Middle 6-week *vs.* Middle Current	**1.52E-03**
	Middle 3-week *vs.* Middle Current	6.02E-01
Delayed treatment effect	End 6-week *vs.* Middle 6-week	**0.0242**
	End Current *vs.* Middle Current	**4.86E-02**
	End 3-week *vs.* Middle 3-week	1.78E-01
	End 6-week *vs.* End Current	0.298
	End 3-week *vs.* End Current	0.845

Table S6. Effects of *period-treatment* on the abundance of benthic taxa that significantly explained community dissimilarity in Figure S8. Significant responses are assigned to seasonal and to treatment effects (*immediate* or *delayed*). Asterisks indicate $P < 0.05$ for *period-treatment*. Short horizontal dashes (~) indicate $P < 0.10$. A positive sign (+) or negative sign (-) indicates if advanced low flow treatment had a positive or negative effect, respectively, on taxa abundance, provided that treatment differences explained post-hoc differences. A combination of positive and negative signs (±) indicates that low flow treatment had positive and negative effects at different times. See conceptual framework on response types in Fig. 1, and Table S1 for how statistical tests connect with each response type.

**Taxa**	**ANOVA response**		
	**Period-Treatment**	**Treatment effect (Immediate/Delayed/Absent)**	**Seasonal effect (Present/Absent)**
Chironomini	*	Delayed (+)	Absent
Epeorus	~	Immediate (-)	Absent
Glossosoma			
Hydroptila	*	Delayed (+)	Present
Muscidae	*	Absent	Absent
Oligochaeta			
Orthocladiinae sensu lato			
Simuliidae	*	Delayed (+)	Present
Tanytarsini			
Turbellaria	~	Delayed (+)	Absent
Baetis	*	Immediate & Delayed (±)	Absent
Tanypodinae sensu lato			
Diphetor			
Lepidostoma	*	Delayed (+)	Present
Micrasema	*	Delayed (-)	Absent
Drunella flavilnea	*	Immediate (-)	Present

Table S7. Pairwise comparisons of benthic Chironomini abundance among *period-treatment* groups. We examined benthic Chironomini abundance via post-hoc pairwise comparisons (*P*-values) corrected by the Benjamini–Hochberg method. A bolded *P*-value indicates the pairwise comparison was significant for that response type. If the *Current* and *3-week* or *6-week* treatments are significant for the same comparison across periods (e.g., *start* vs. *middle*) then *P*-values are bolded for *seasonal* effects but not for *immediate* or *delayed* treatment effects, as that would indicate similar change over time regardless of treatment. See conceptual framework on response types in Fig. 1, and Table S1 for how statistical tests connect with each response type.

**Response type**	**Relevant comparisons**	***P*-value**
Seasonal effect	Middle 6-week *vs.* Start 6-week	0.672
	Middle Current *vs.* Start Current	0.545
	End 6-week *vs.* Middle 6-week	0.000519
	End Current *vs.* Middle Current	0.113
Immediate treatment effect	Middle 6-week *vs.* Start 6-week	0.672
	Middle Current *vs.* Start Current	0.545
	Middle 6-week *vs.* Middle Current	0.862
Delayed treatment effect	End 6-week *vs.* Middle 6-week	**0.000519**
	End Current *vs.* Middle Current	0.113
	End 6-week *vs.* End Current	0.0506

Table S8. Pairwise comparisons of benthic Hydroptila abundance among *period-treatment* groups. Benthic *Hydroptila* abundance post-hoc pairwise comparisons (*P*-values) corrected by the Benjamini–Hochberg method. A bolded *P*-value indicates the pairwise comparison was significant for that response type. If the *Current* and *3-week* or *6-week* treatments are significant for the same comparison across periods (e.g., *start* vs. *middle*) then *P*-values are bolded for *seasonal* effects but not for *immediate* or *delayed* treatment effects, as that would indicate similar change over time regardless of treatment. See conceptual framework on response types in Fig. 1, and Table S1 for how statistical tests connect with each response type.

Response type	Relevant comparisons	*P*-value
Seasonal effect	Middle 6-week *vs.* Start 6-week	0.238
	Middle Current *vs.* Start Current	0.985
	End 6-week *vs.* Middle 6-week	**1.54E-09**
	End Current *vs.* Middle Current	**0.00459**
Immediate treatment effect	Middle 6-week *vs.* Start 6-week	0.238
	Middle Current *vs.* Start Current	0.985
	Middle 6-week *vs.* Middle Current	0.238
Delayed treatment effect	End 6-week *vs.* Middle 6-week	1.54E-09
	End Current *vs.* Middle Current	0.00459
	End 6-week *vs.* End Current	**0.0000721**

Table S9. Pairwise comparisons of benthic Simuliidae abundance among *period-treatment* groups. Benthic Simuliidae abundance post-hoc pairwise comparisons (*P*-values) corrected by the Benjamini–Hochberg method. A bolded *P*-value indicates the pairwise comparison was significant for that response type. If the *Current* and *3-week* or *6-week* treatments are significant for the same comparison across periods (e.g., *start* vs. *middle*) then *P*-values are bolded for *seasonal* effects but not for *immediate* or *delayed* treatment effects, as that would indicate similar change over time regardless of treatment. See conceptual framework on response types in Fig. 1, and Table S1 for how statistical tests connect with each response type.

Response type	Relevant comparisons	*P*-value
Seasonal effect	Middle 6-week *vs.* Start 6-week	0.8
	Middle Current *vs.* Start Current	0.9
	End 6-week *vs.* Middle 6-week	**0.0000146**
	End Current *vs.* Middle Current	**0.0197**
Immediate treatment effect	Middle 6-week *vs.* Start 6-week	0.8
	Middle Current *vs.* Start Current	0.9
	Middle 6-week *vs.* Middle Current	0.8
Delayed treatment effect	End 6-week *vs.* Middle 6-week	0.0000146
	End Current *vs.* Middle Current	0.0197
	End 6-week *vs.* End Current	**0.0152**

Table S10. Pairwise comparisons of benthic *Micrasema* abundance among *period-treatment* groups. Benthic *Micrasema* abundance post-hoc pairwise comparisons (*P*-values) corrected by the Benjamini–Hochberg method. A bolded *P*-value indicates the pairwise comparison was significant for that response type. If the *Current* and *3-week* or *6-week* treatments are significant for the same comparison across periods (e.g., *start* vs. *middle*) then *P*-values are bolded for *seasonal* effects but not for *immediate* or *delayed* treatment effects, as that would indicate similar change over time regardless of treatment. See conceptual framework on response types in Fig. 1, and Table S1 for how statistical tests connect with each response type.

Response type	Relevant comparisons	*P*-value
Seasonal effect	Middle 6-week *vs.* Start 6-week	0.837
	Middle Current *vs.* Start Current	0.347
	End 6-week *vs.* Middle 6-week	0.0169
	End Current *vs.* Middle Current	0.28
Immediate treatment effect	Middle 6-week *vs.* Start 6-week	0.837
	Middle Current *vs.* Start Current	0.347
	Middle 6-week *vs.* Middle Current	0.341
Delayed treatment effect	End 6-week *vs.* Middle 6-week	**0.0169**
	End Current *vs.* Middle Current	0.28
	End 6-week *vs.* End Current	0.516

Table S11. Pairwise comparisons of benthic stream invertebrate NMDS community composition among *period-treatment* groups. A PERMANOVA test was first conducted with *period-treatment* as a fixed effect and it was found to significantly explain benthic community composition (pseudo-$F_{5,35}$ = 2.571, P < 0.001). Benthic stream invertebrate NMDS community post-hoc pairwise comparisons (P-values) corrected by the Benjamini–Hochberg method. A bolded P-value indicates the pairwise comparison was significant for that response type. If the *Current* and *3-week* or *6-week* treatments are significant for the same comparison across periods (e.g., *start* vs. *middle*) then P-values are bolded for *seasonal* effects but not for *immediate* or *delayed* treatment effects, as that would indicate similar change over time regardless of treatment. See conceptual framework on response types in Fig. 1, and Table S1 for how statistical tests connect with each response type.

Response type	Relevant comparisons	***P*-value**
Seasonal effect	Middle 6-week *vs.* Start 6-week	0.105
	Middle Current *vs.* Start Current	0.171
	End 6-week *vs.* Middle 6-week	0.0188
	End Current *vs.* Middle Current	0.209
Immediate treatment effect	Middle 6-week *vs.* Start 6-week	0.105
	Middle Current *vs.* Start Current	0.171
	Middle 6-week *vs.* Middle Current	0.464
Delayed treatment effect	End 6-week *vs.* Middle 6-week	**0.0188**
	End Current *vs.* Middle Current	0.209
	End 6-week *vs.* End Current	0.171

Table S12. The effects of *period-treatment* on the abundance of emergent taxa that significantly explain community dissimilarity ($P \leq 0.002$) in Figure S8. Significant responses are determined to be due to seasonal or treatment effects. Asterisks indicate $P < 0.05$ for *period-treatment*. A positive sign (+) indicates that low flow treatment had a positive effect on taxa abundance, provided that treatment differences explained post-hoc differences. See conceptual framework on response types in Fig. 1, and Table S1 for how statistical tests connect with each response type.

**Taxa**	**ANOVA response**		
	**Period-Treatment**	**Treatment effect (Immediate/Delayed/Absent)**	**Seasonal effect (Present/Absent)**
Chironominae	*	Immediate & Delayed (+)	Present
Prodiamesinae	*	Immediate (+)	Absent
Orthocladiinae	*	Absent	Absent

Table S13. Pairwise comparisons of emergent Chironominae abundance among *period-treatment* groups. Emergent Chironominae abundance post-hoc pairwise comparisons (*P*-values) corrected by the Benjamini–Hochberg method. A bolded *P*-value indicates the pairwise comparison was significant for that response type. If the *Current* and *3-week* or *6-week* treatments are significant for the same comparison across periods (e.g., *start* vs. *middle*), then *P*-values are bolded for *seasonal* effects but not for *immediate* or *delayed* treatment effects, as that would indicate similar change over time regardless of treatment. See conceptual framework on response types in Fig. 1, and Table S1 for how statistical tests connect with each response type.

**Response type**	**Relevant comparisons**	***P*-value**
Seasonal effect	Middle 6-week *vs.* Start 6-week	**3.75E-05**
	Middle Current *vs.* Start Current	**4.07E-03**
	Middle 3-week *vs.* Start 3-week	**4.39E-03**
	End 6-week *vs.* Middle 6-week	0.755
	End Current *vs.* Middle Current	1.48E-01
	End 3-week *vs.* Middle 3-week	2.99E-02
Immediate treatment effect	Middle 6-week *vs.* Start 6-week	3.75E-05
	Middle Current *vs.* Start Current	4.07E-03
	Middle 3-week *vs.* Start 3-week	4.39E-03
	Middle 6-week *vs.* Middle Current	**8.18E-03**
	Middle 3-week *vs.* Middle Current	9.32E-01
Delayed treatment effect	End 6-week *vs.* Middle 6-week	0.755
	End Current *vs.* Middle Current	1.48E-01
	End 3-week *vs.* Middle 3-week	**2.99E-02**
	End 6-week *vs.* End Current	0.755
	End 3-week *vs.* End Current	0.584

Table S14. Pairwise comparisons of emergent stream invertebrate NMDS community composition among *period-treatment* groups. A PERMANOVA test was first conducted with *period-treatment* as a fixed effect and it was found to significantly explain emergent community composition (pseudo-$F_{8,89}$ = 5.7277, $P < 0.001$). Emergent stream invertebrate NMDS community post-hoc pairwise comparisons (*P*-values) corrected by the Benjamini–Hochberg method. A bolded *P*-value indicates the pairwise comparison was significant for that response type. If the *Current* and *3-week* or *6-week* treatments are significant for the same comparison across periods (e.g., *start* vs. *middle*) then *P*-values are bolded for *seasonal* effects but not for *immediate* or *delayed* treatment effects, as that would indicate similar change over time regardless of treatment. See conceptual framework on response types in Fig. 1, and Table S1 for how statistical tests connect with each response type.

Response type	Relevant comparisons	*P*-value
Seasonal effect	Middle 6-week *vs.* Start 6-week	**0.00257**
	Middle Current *vs.* Start Current	**0.00257**
	Middle 3-week *vs.* Start 3-week	**0.00257**
	End 6-week *vs.* Middle 6-week	0.38
	End Current *vs.* Middle Current	0.197
	End 3-week *vs.* Middle 3-week	0.0208
Immediate treatment effect	Middle 6-week *vs.* Start 6-week	0.00257
	Middle Current *vs.* Start Current	0.00257
	Middle 3-week *vs.* Start 3-week	0.00257
	Middle 6-week *vs.* Middle Current	**0.0394**
	Middle 3-week *vs.* Middle Current	0.883
Delayed treatment effect	End 6-week *vs.* Middle 6-week	0.38
	End Current *vs.* Middle Current	0.197
	End 3-week *vs.* Middle 3-week	**0.0208**
	End 6-week *vs.* End Current	0.883
	End 3-week *vs.* End Current	0.532

Table S15 Assessment of potential Brewer's Blackbird effects on benthic and emergent macroinvertebrate communities, using a Before-After-Control-Impact (BACI) design. We tested if benthic community composition and structure changed immediately after blackbird arrival in channels that were visited relative to channels that were not visited. The interaction term of treatment*time (time being binary: pre vs post-bird presence) was not statistically significant when examining either benthic or emergent invertebrate abundance, richness, or composition, denoting no significant bird effects. Degrees of freedom (df) lists the df for the treatment*time interaction first, then the df of residuals.

**Response variable**	**df**	**F statistic**	***P*-value for treatment*time**
Benthic abundance	1, 30	0.238	0.629
Benthic species richness	1, 30	1.649	0.209
Benthic community composition	1, 32	0.957	0.438
Emergent abundance	2, 82	0.269	0.765
Emergent species richness	2, 82	0.62	0.540
Emergent community composition	2, 84	0.716	0.693

SI References

1. K. Reich, *et al.*, Climate Change in the Sierra Nevada: California's Water Future. *UCLA Cent. Clim. Sci.* (2018).

2. J. R. Blaszczak, *et al.*, Extent, patterns, and drivers of hypoxia in the world's streams and rivers. *Limnol. Oceanogr. Lett.* **8**, 453–463 (2023).

3. A. V. Nebeker, Effect of Low Oxygen Concentration on Survival and Emergence of Aquatic Insects. *Trans. Am. Fish. Soc.* **101**, 675–679 (1972).

4. S. R. P. -J. Ross, O. L. Petchey, T. Sasaki, D. W. Armitage, How to measure response diversity. *Methods Ecol. Evol.* **14**, 1150–1167 (2023).
